# Definitions, epidemiology, and outcomes of persistent/chronic critical illness: a scoping review for translation to clinical practice

**DOI:** 10.1186/s13054-024-05215-4

**Published:** 2024-12-28

**Authors:** Hiroyuki Ohbe, Kasumi Satoh, Takaaki Totoki, Atsushi Tanikawa, Kasumi Shirasaki, Yoshihide Kuribayashi, Miku Tamura, Yudai Takatani, Hiroyasu Ishikura, Kensuke Nakamura, J-STAD (JAPAN Sepsis Treatment and Diagnosis) Study Group

**Affiliations:** 1 Department of Emergency and Critical Care Medicine, Tohoku University Hospital, 1-1 Seiryo-machi, Aoba-ku, Sendai, 980-8574 Japan; 2 Department of Clinical Epidemiology and Health Economics, School of Public Health, The University of Tokyo, 7-3-1 Hongo, Bunkyo-ku, Tokyo, 113-0033 Japan; 3 Department of Emergency and Critical Care Medicine, Akita University Graduate School of Medicine, 1-1-1 Hondo, Akita, 010-8543 Japan; 4 Department of Emergency and Critical Care Medicine, Osaka Medical and Pharmaceutical University, 2-7 Daigakumachi, Takatsuki, Osaka 569-8686 Japan; 5 Department of Emergency and Critical Care Medicine, St. Luke’s International Hospital, 9-1 Akashicho, Chuo-ku, Tokyo, 104-8560 Japan; 6 Department of Emergency and Disaster Medicine, Kanazawa University Hospital, 13, 1-1 Takara-Machi, Kanazawa 920-8640, Aoba-ku, Sendai, 980-8574 Japan; 7 Department of Anesthesiology and Intensive Care Medicine, Faculty of Medicine, Oita University, 1-1 Idaigaoka, Hasamacho, Yufu, Oita, 879-5593 Japan; 8 Department of Pharmacy, Funabashi Municipal Medical Center, 1-21-1 Kanasugi, Funabashi city, Chiba, Japan; 9 Department of Primary Care and Emergency Medicine, Kyoto University Hospital, 54 Shogoin-Kawahara-cho, Sakyo-ku, Kyoto, 606-8507 Japan; 10 Department of Emergency and Critical Care Center, Rakuwakai Otowa Hospital, 2 Otowachinji-cho, Yamashina-ku, Kyoto, 607-8062 Japan; 11 Department of Critical Care Medicine, Yokohama City University Hospital, 3-9 Fukuura, Kanazawa-ku, Yokohama, Kanagawa 236-0004 Japan

**Keywords:** Intensive care unit, Persistent critical illness, Chronic critical illness, Scoping review

## Abstract

**Background:**

Medical advances in intensive care units (ICUs) have resulted in the emergence of a new patient population—those who survive the initial acute phase of critical illness, but require prolonged ICU stays and develop chronic critical symptoms. This condition, often termed Persistent Critical Illness (PerCI) or Chronic Critical Illness (CCI), remains poorly understood and inconsistently reported across studies, resulting in a lack of clinical practice use. This scoping review aims to systematically review and synthesize the existing literature on PerCI/CCI, with a focus on definitions, epidemiology, and outcomes for its translation to clinical practice.

**Methods:**

A scoping review was conducted using MEDLINE and Scopus, adhering to the PRISMA-ScR guidelines. Peer-reviewed original research articles published until May 31, 2024 that described adult PerCI/CCI in their definitions of patient populations, covariates, and outcomes were included. Data on definitions, epidemiology, and outcomes were extracted by a data charting process from eligible studies and synthesized.

**Results:**

Ninety-nine studies met the inclusion criteria. Of these studies, 64 used the term CCI, 18 used PerCI, and 17 used other terms. CCI definitions showed greater variability, while PerCI definitions remained relatively consistent, with an ICU stay ≥ 14 days for CCI and ≥ 10 days for PerCI being the most common. A meta-analysis of the prevalence of PerCI/CCI among the denominators of “all ICU patients”, “sepsis”, “trauma”, and “COVID-19” showed 11% (95% confidence interval 10–12%), 28% (22–34%), 24% (15–33%), and 35% (20–50%), respectively. A meta-analysis of in-hospital mortality was 27% (26–29%) and that of one-year mortality was 45% (32–58%). Meta-analyses of the prevalence of CCI and PerCI showed 17% (16–18%) and 18% (16–20%), respectively, and those for in-hospital mortality were 28% (26–30%) and 26% (24–29%), respectively. Functional outcomes were generally poor, with many survivors requiring long-term care.

**Conclusions:**

This scoping review synthesized many studies on PerCI/CCI, highlighting the serious impact of PerCI/CCI on patients’ long-term outcomes. The results obtained underscore the need for consistent terminology with high-quality research for PerCI/CCI. The results obtained provide important information to be used in discussions with patients and families regarding prognosis and care options.

**Graphical abstract:**

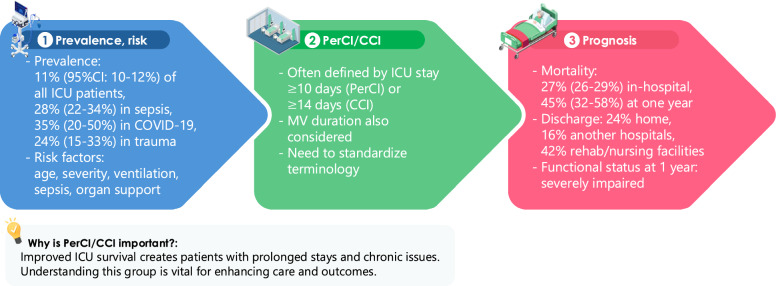

**Supplementary Information:**

The online version contains supplementary material available at 10.1186/s13054-024-05215-4.

## Introduction

Advances in medical technology have improved survival rates in intensive care units (ICU), which has resulted in the emergence of a new patient population—those who survive the initial acute phase of critical illness, but require prolonged ICU stays and develop chronic critical symptoms [1, 2]. These patients are often termed persistent critical illness (PerCI), chronic critical illness (CCI), post-intensive care syndrome (PICS), and persistent inflammatory, immunosuppressive, and catabolic syndrome (PIICS). There are many overlapping features among these terms, and each has its specific focus [3]. Among them, the terms PerCI and CCI focus on the distinction between “acute” and “chronic/persistent”. Many studies have adopted PerCI and CCI because of the simplicity of their definitions, and they also do not require detailed assessments for the physical, cognitive, and mental health domains of the PICS definition or the measurement of biomarkers for the pathophysiological mechanisms of the PIICS definition [3]. However, it is debatable whether there is a clinically meaningful difference between CCI and PerCI [3].

PerCI/CCI is a devastating condition for patients and their families, with poor long-term mortality and morbidity, and it has recently become a serious and growing issue for healthcare systems worldwide [4, 5]. Despite its importance, the prevalence, definitions, risk factors, prognoses, and outcomes of these patients remain unclear and are inconsistently reported across studies. While several reviews have been conducted on this topic, many are outdated or lack the methodological rigor found in scoping or systematic reviews that comprehensively search and assess original articles [2, 3, 6–13]. Therefore, there are gaps in the literature and an incomplete understanding of PerCI/CCI. The concept of PerCI/CCI is not currently used in clinical practice due to the lack of organized information; however, it is an important health issue that needs to be recognized and requires interventions in clinical practice.

Therefore, this scoping review aims to systematically review and synthesize the existing literature on PerCI/CCI, with a focus on the definitions, prevalence, risk factors, and outcomes associated with these conditions. Regarding its translation to clinical practice, we seek to provide a comprehensive overview that will inform both clinical practice and future research, as well as support the provision of critical information to patients and their families who face these challenging diagnoses.

## Methods

### Protocol and registration

This scoping review is reported in line with the Preferred Reporting Items for Systematic Reviews and Meta-Analyses extension for Scoping Reviews (PRISMA-ScR) guidelines (the checklist is shown in Supplementary Material) [14]. The protocol was registered with the UMIN Clinical Trials Registry (UMIN-CTR) a priori (UMIN000054545) on June 1, 2024. The question examined in this scoping review was as follows: “What are the definitions, epidemiology, and outcomes of adults with PerCI/CCI as described in original research articles?”.

### Eligibility criteria

Peer-reviewed original research articles were included if they were published by May 31, 2024, written in English, involved adult participants, and used terminology to describe PerCI/CCI in the definitions of patient populations, covariates, and outcomes, with definitions explicitly stated in the article. Three reviewers (HO, TT, and KN) reviewed terms for PerCI/CCI and adopted the following five terms: “persistent critical illness”, “chronic critical illness”, “chronic critically ill”, “chronically critically ill”, and “prolonged critical illness”. Studies were excluded if they were unpublished, preprints, conference abstracts without subsequent study publication, studies not in English, review articles, animal studies, or studies on children.

### Search strategy

We used the bibliographic databases of MEDLINE (via Ovid) and Scopus. The search was performed on June 1, 2024 for MEDLINE and on November 10, 2024 for Scopus and full search strategies may be found in Supplementary Material. The final search results were exported into Rayyan [15], and duplicates were removed by the duplicate resolution system provided by Rayyan and two independent reviewers (HO and TT). In the first screening, two investigators (HO and TT) independently assessed eligibility by screening the study titles and abstracts after removing duplicates. Five investigators (HO, TT, AT, MT, and KN) then evaluated eligibility based on full-text manuscripts of all publications identified in the first screening. We resolved any disagreement through discussions under the supervision of a senior investigator (HO).

### Data charting process

Regarding each section on definitions, epidemiology (prevalence, characteristics, and risk factors), and outcomes (prognosis, trajectory, and functional outcome), data-charting forms were jointly developed by two reviewers (HO and AT, KS and YK, and MT and YT, respectively) to identify which variables to extract. The two reviewers independently charted the data, discussed the results, and continuously updated the data-charting form for each section in an iterative process [16]. Any disagreements were resolved through discussions between the two reviewers or further adjudication by supervision from one senior investigator (HO). Data charting was implemented using Excel software.

#### Synthesis of results

We created tables to show results for abstracted data. Meta-analyses of prevalence, age, acute physiology and chronic health evaluation (APACHE) II scores, in-hospital mortality, and one-year mortality stratified by the terms for PerCI/CCI were performed because these data were important and there was a sufficient number of articles reporting these data. Meta-analyses were conducted with a random-effects model using the method of DerSimonian and Laird, with the estimate of heterogeneity being taken from the inverse-variance fixed-effect model. A test of whether the summary effect measure was equal to zero was performed and heterogeneity was also quantified using the I^2^ statistic (≥ 50% indicates moderate heterogeneity). These meta-analyses and the creation of forest plot graphs were performed using “metaprop” for binary variables and “metan” for continuous variables in STATA command [17, 18]. When median values were provided for continuous variables, they were used as the mean, and when the interquartile range (IQR) was provided, the standard deviation was calculated from IQR/1.35 [19]. Subgroup meta-analyses stratified by “persistent critical illness” and “chronic critical illness” and sensitivity meta-analyses stratified by seven continents were also conducted on prevalence and in-hospital mortality. Serial studies from the same cohort with overlapping observation periods were identified. To avoid double-counting, only the study with the largest population or the most recent study reporting each variable was included in meta-analyses.

## Results

### Selection of sources of evidence

**Fig. 1:**
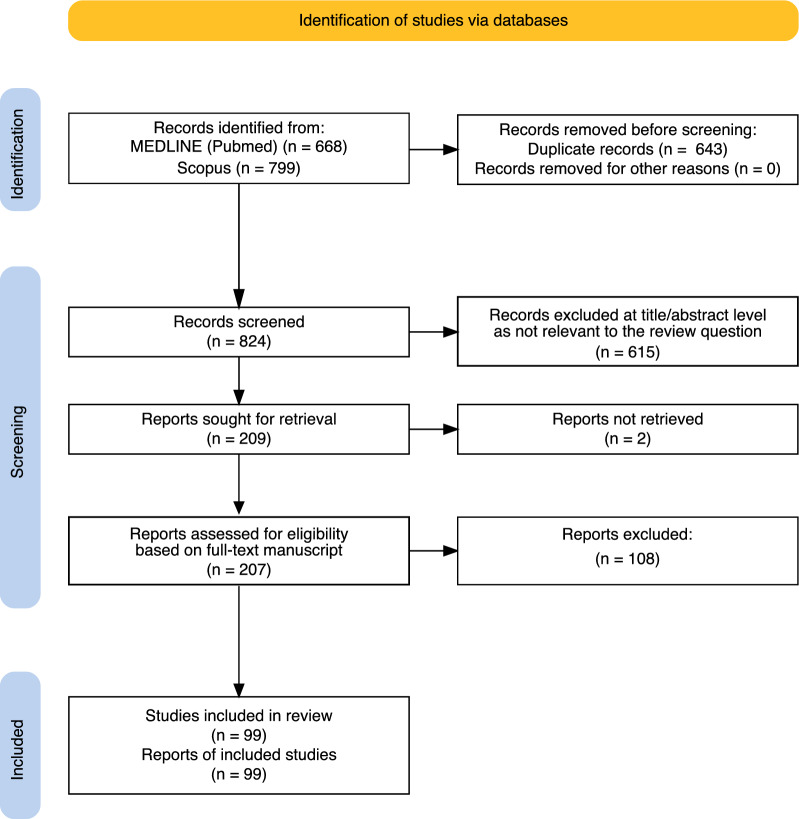
Selection of sources of evidence

After the removal of duplicates, 824 citations were identified from searches of electronic databases (Fig. 1). Based on the title and abstract, 615 were excluded, with 209 full-text articles being retrieved and assessed for eligibility. We excluded 2 studies because we were unable to retrieve them. A further 108 studies were excluded because they did not explicitly state the definitions of PerCI/CCI. Therefore, the remaining 99 studies were considered eligible for this review [20–118].

### Characteristics of sources of evidence

The studies’ author, year, country, type of study, and terminology of PerCI/CCI are presented in Supplemental Table 1. Of the 99 studies, 64 used the term “chronic critical illness”, 18 used “persistent critical illness”, 9 used “chronic critically ill”, 6 used “prolonged critical illness”, and 2 used “chronically critically ill”. The terms “chronic critically ill”, “prolonged critical illness”, and “chronic critically ill” were mainly used in the 2000s, while the terms “chronic critical illness” and “persistent critical illness” have been used in recent years, with many publications after 2019 (Supplemental Fig. 1). Serial studies were identified from 6 cohorts and presented in Supplemental Table 2.

### Definition

A summary of the definition of PerCI/CCI is shown in Table 1 and Supplemental Table 1. Although the definition varies widely, most studies used the ICU or mechanical ventilation (MV) duration in the definition. Of the 64 studies that used the term “chronic critical illness”, 49 (76%) involved ICU stays with a specific duration (≥ 5 to ≥ 21 days) with persistent organ dysfunction or additional conditions, 12 (19%) involved MV for a specific duration (≥ 7 to ≥ 21 days) or tracheostomy, and 3 (5%) were other criteria. The most common definition was an ICU stay ≥ 14 days with persistent organ dysfunction, accounting for 50% (n = 32/64). Of the 18 studies that used the term “persistent critical illness”, all 18 focused on ICU stays and the most common criterion was an ICU stay ≥ 10 days, accounting for 83% (n = 15/18). Definitions of the terms “chronic critically ill”, “prolonged critical illness”, and “chronically critically ill” varied across studies, with no duplicated definition being found among studies.

**Table 1:** Summary of definitions for PerCI/CCI

Terminology	Definition*	Number	Percentage (%)
Chronic critical illness (CCI)		64	
	ICU stay ≥ 14 days with persistent organ dysfunction	32	50
	ICU stay ≥ 8 days and one of six eligible clinical conditions	5	8
	MV ≥ 7 days	4	6
	ICU stay > 14 days	2	3
	ICU stay ≥ 21 days	2	3
	MV ≥ 14 days or tracheostomy	2	3
Persistent critical illness (PerCI)		18	
	ICU stay ≥ 10 days	15	83

*PerCI* persistent critical illness; *CCI* chronic critical illness; *ICU* Intensive care unit; *MV* mechanical ventilation

*Unduplicated definitions are not listed

### Prevalence

**Fig. 2:**
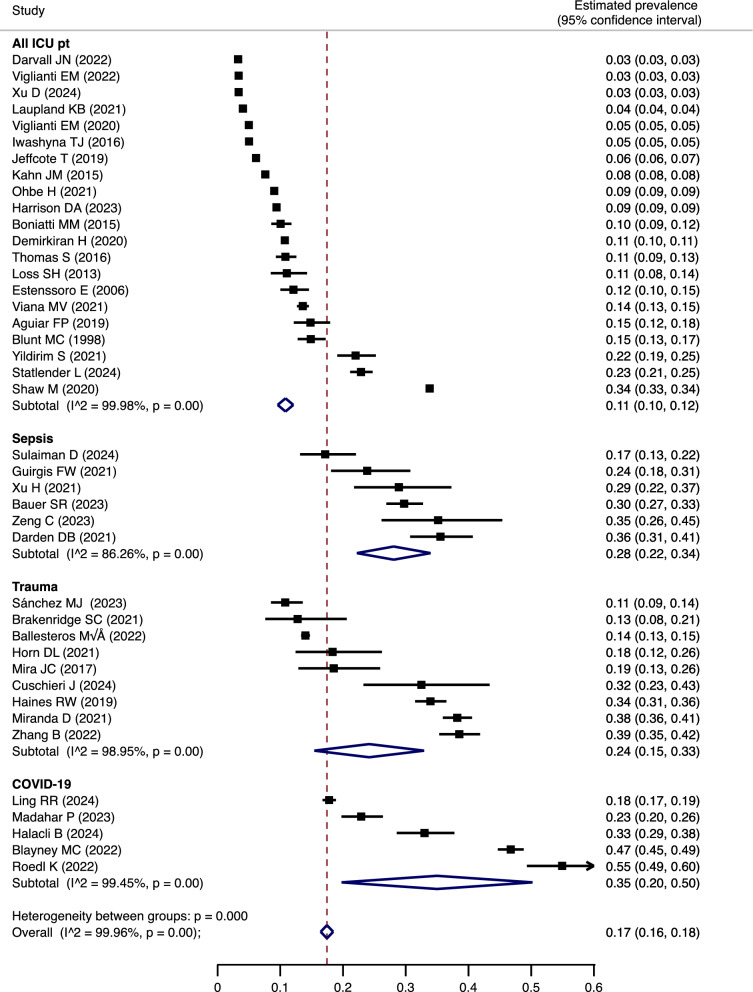
Meta-analysis for prevalence of PerCI/CCI. The summary statistics (diamonds) for each stratum and all studies overall are the results of a random effects model. *PerCI* persistent critical illness; *CCI* chronic critical illness; *ICU* intensive care unit; *COVID-19* corona virus infection disease 2019

Seventy studies reported the prevalence of PerCI/CCI; however, denominators for prevalence calculations varied widely (Supplemental Table 3). After discussions on the data charting process, we abstracted the prevalence of PerCI/CCI stratified by the denominators of “all ICU patients”, “sepsis”, “trauma”, “corona virus disease 2019 (COVID-19)”, and “Other”. We then excluded 11 studies categorized by “Other” and 18 serial studies and conducted meta-analyses. The overall estimated prevalence of PerCI/CCI in 41 studies regardless of the denominator was 17% (95% confidence interval [CI] 16–18%) (Fig. 2). The estimated prevalence among the denominators of “all ICU patients”, “sepsis”, “trauma”, and “COVID-19” were 11% (10–12%), 28% (22–34%), 24% (15–33%), and 35% (20–50%), respectively. Subgroup meta-analyses showed that the overall estimated prevalence among 23 studies using definitions of the terms “chronic critical illness” was 17% (16–18%), while that among 13 studies using “persistent critical illness” was 18% (16–20%) (Supplemental Figs. 2, 3). The estimated prevalence among denominators of “all ICU patients” were 10% (9–11%) and 11% (9–14%) for the terms “chronic critical illness” and “persistent critical illness”, respectively. Sensitivity meta-analyses showed that the overall estimated prevalence in North America, Europe, Asia, and South America were 17% (16–19%), 20% (18–22%), 15% (13–17%), and 13% (11–15%), respectively (Supplemental Fig. 4).

### Characteristics and risk factors

Meta-analyses of 55 studies showed that overall estimated mean age was 62.2 (60.7–63.6), while those for the terms “chronic critical illness” and “persistent critical illness” was 63.4 (61.4–65.5) and 60.5 (58.4–62.6), respectively (Supplemental Fig. 5). In most studies, males were predominant (60–70%) and they often had high rates of MV (> 90%) and tracheostomy (approximately 20%) (Supplemental Table 3). Meta-analyses of 29 studies showed that the overall estimated mean APACHE II score was 18.1 (17.3–18.9), while those for the terms “chronic critical illness” and “persistent critical illness” were 19.9 (18.3–21.5) and 17.7 (16.2–19.2), respectively (Supplemental Fig. 6). None of the studies that examined COVID-19 provided a description of long-COVID.

The most commonly reported risk factors for “chronic critical illness” included an older age (10 studies), higher organ failure score (e.g., APACHE II and sequential organ failure assessment) (8 studies), sepsis (4 studies), MV (4 studies), and injury severity score (4 studies) (Table 2 and Supplemental Table 4). All reported risk factors for “persistent critical illness” had no overlap. Few studies reported risk factors for “chronic critically ill”, “chronically critically ill”, and “prolonged critical illness”. Potential biomarkers for the development of PerCI/CCI were included among risk factors, with two studies reporting PT -INR and albumin and one study using GLP-1, ApoA-I, LDL-C, SIRT2, D7-sPD-L1, the urea-to-creatinine ratio, lymphocytes, the monocyte-to-lymphocyte ratio, and glucose.

**Table 2:** Summary of risk factors for “chronic critical illness”

Terminology	Risk factors*	Number
Chronic critical illness (CCI)	Older age	10
	Organ failure score defined by SOFA, APACHE, or Denver MOF	8
	Sepsis	4
	MV	4
	Injury severity score	4
	Shock	3
	Vasopressor use	3
	Body mass index	2
	Charlson Comorbidity Index	2
	Glasgow coma scale	2
	Albumin	2
	PT-INR	2

*CCI* chronic critical illness; *ICU* intensive care unit; *MV* mechanical ventilation; *SOFA* sequential organ failure assessment; *APACHE* acute physiology and chronic health evaluation; *MOF* multiple organ failure; *PT-INR* prothrombin time-international normalized ratio

*Unduplicated risk factors are not listed

### Prognosis, trajectory, and functional outcomes

**Fig. 3:**
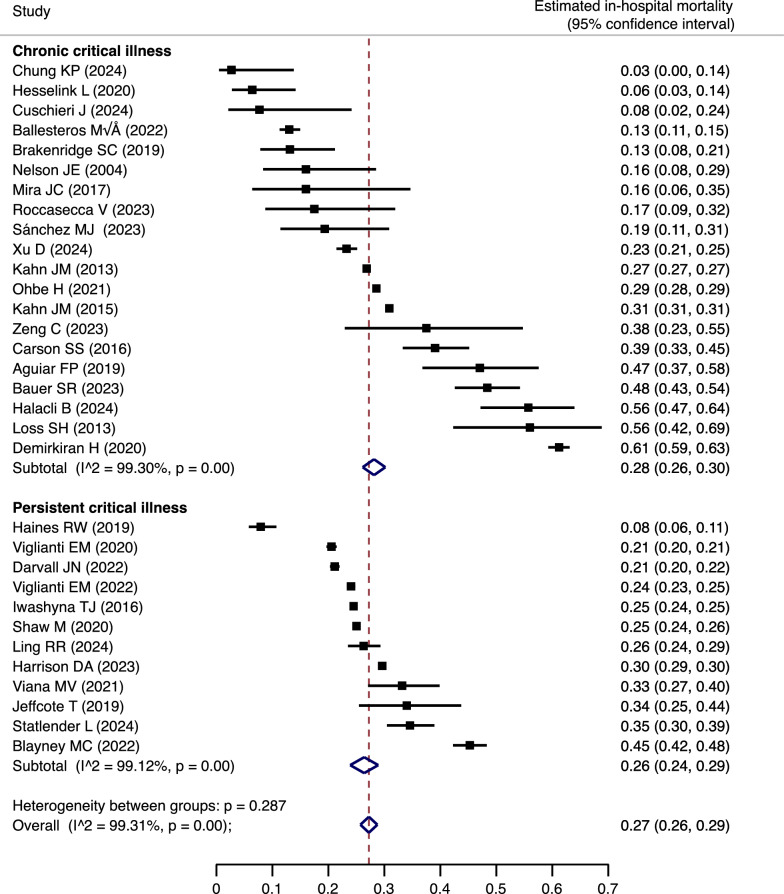
Meta-analysis for in-hospital mortality of PerCI/CCI. The summary statistics (diamonds) for each stratum and all studies overall are the results of a random effects model. *PerCI* persistent critical illness; *CCI* chronic critical illness

Forty-five studies reported in-hospital mortality (Supplemental Table 5), and since “chronic critically ill”, “chronically critically ill”, and “prolonged critical illness” were reported by one study each, a meta-analysis of “chronic critical illness” and “persistent critical illness” was conducted. After the exclusion of 9 serial studies, the overall estimated in-hospital mortality of PerCI/CCI in 32 studies was 27% (95% CI 26–29%). Estimated in-hospital mortality was 28% (26–30%) for “chronic critical illness” and 26% (24–29%) for “persistent critical illness”, with no heterogeneity between groups (p = 0.287) (Table 3 and Fig. 3). Sensitivity meta-analyses showed that estimated in-hospital mortality in North America, Europe, Asia, and South America were 28% (26–30%), 22% (18–26%), 43% (16–69%), and 50% (42–59%), respectively (Supplemental Fig. 7). A meta-analysis of “chronic critical illness” in four studies showed that estimated one-year mortality was 45% (32–58%) (Supplemental Fig. 8).

**Table 3:** Summary of outcomes for PerCI/CCI

Outcomes	Number of studies	Results of meta-analyses (95%CI)	Median of studies	Min–max
Chronic critical illness (CCI)				
In-hospital mortality	20	28% (26–30%)	–	3–61%
One-year mortality	4	45% (32–58%)	–	27–62%
Median length of ICU, days	27	–	21	13–73
Median length of stay, days	6	–	43	31–47
Median length of MV, days	13	–	20	6–53
Discharge destinations				
Home	6	–	24%	11–35%
Another hospital	6	–	16%	4–40%
Rehabilitation facilities	5	–	26%	3–46%
Nursing facilities	6	–	16%	1–39%
Hospice	3	–	6%	2–9%
Functional outcome				
Zubrod scores at 12 months	7	–	3.5	3.2–3.5
EQ-5D utility at 12 months	2	–	0.71	0.55–0.86
S BBP scores at 12 months	3	–	5.0	3.1–5.3
Persistent critical illness (PerCI)				
In-hospital mortality	12	26% (24–29%)	–	8–45%
Median length of ICU, days	15	–	17	4–28
Median length of stay, days	3	–	28	25–32
Median length of MV, days	2	–	17.5	16–19
Discharge destinations				
Home	3	–	43%	18–47%
Another hospital	2	–	19.5%	8–31%
Rehabilitation facilities	3	–	12%	11–38%

*PerCI* persistent critical illness; *CCI* chronic critical illness; *CI* confidence intervals; *ICU* intensive care unit; *MV* mechanical ventilation; *EQ-5D* EuroQol five-dimensional; *SPPB* Short Physical Performance Battery

The median of median durations of ICU stay, hospital stay, and MV were 21, 43, and 20 days, respectively (Table 3). The median percentages of median home discharge, transfers to another hospital, rehabilitation facilities, nursing facilities, and hospice were 24, 16, 26, 16, and 6%, respectively (Table 3 and Supplemental Table 6).

Functional outcomes reported across various studies indicated that the mean Zubrod Performance Score at 12 months was consistently between 3.2 and 3.5, reflecting patients capable of only limited self-care and confined to bed or a chair 50% or more of waking hours (Table 3 and Supplemental Table 7). Other metrics, such as the Short Physical Performance Battery score, ranged between 3.1 and 5.3, and EuroQol five-dimensional utility scores at 12 months ranged between 0.55 and 0.86, highlighting worsened levels of physical performance and quality of life.

## Discussion

This review underscores the variability in definitions and terminologies used to describe PerCI/CCI across studies. Over the past decade, the terms CCI and PerCI have become more widely accepted and utilized, rather than “chronic critically ill”, “prolonged critical illness”, and “chronically critically ill”. While CCI shows significant variability in its definition, the definition of PerCI has remained relatively consistent. CCI and PerCI both focus primarily on the duration of the ICU stay, with critical time points often differing only slightly, such as 10 or 14 days. In addition, this review found no clinically meaningful differences in prevalence and mortality between patients with CCI and PerCI. Given that recent increasing trend and relatively uniform definition of PerCI, it may be worth considering standardizing the use of the term PerCI for these conditions to enhance comparability across studies and improve the consistency in research and care strategies and [3].

Although PerCI/CCI requires standardization, PICS and PIICS serve distinct purposes from PerCI/CCI. PICS focuses on the long-term sequelae of ICU survivors, encompassing physical, mental, and cognitive impairments to drive interventions that minimize these disabilities [9]. PIICS targets persistent inflammation, immunosuppression, and catabolism to identify the underlying mechanisms and select appropriate therapeutic interventions [3]. While the use of these terms needs to continue where appropriate, further research is needed to explore whether the clinical and metabolic definitions overlap.

**Fig. 4:**
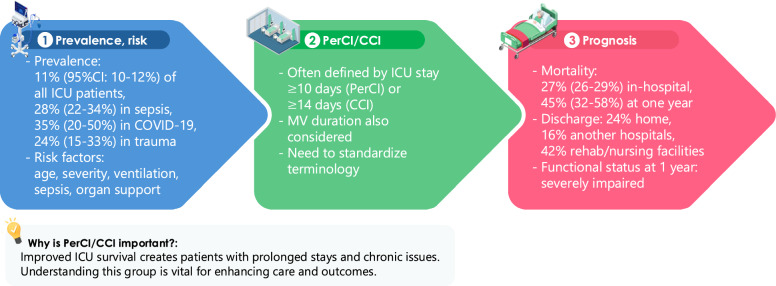
Infographic of a comprehensive summary of this review for translation to clinical practice. CI, confidence interval; ICU, intensive care unit; COVID-19, corona virus infection disease 2019; *PerCI* persistent critical illness; *CCI* chronic critical illness; *MV* mechanical ventilation

**Fig. 5:**
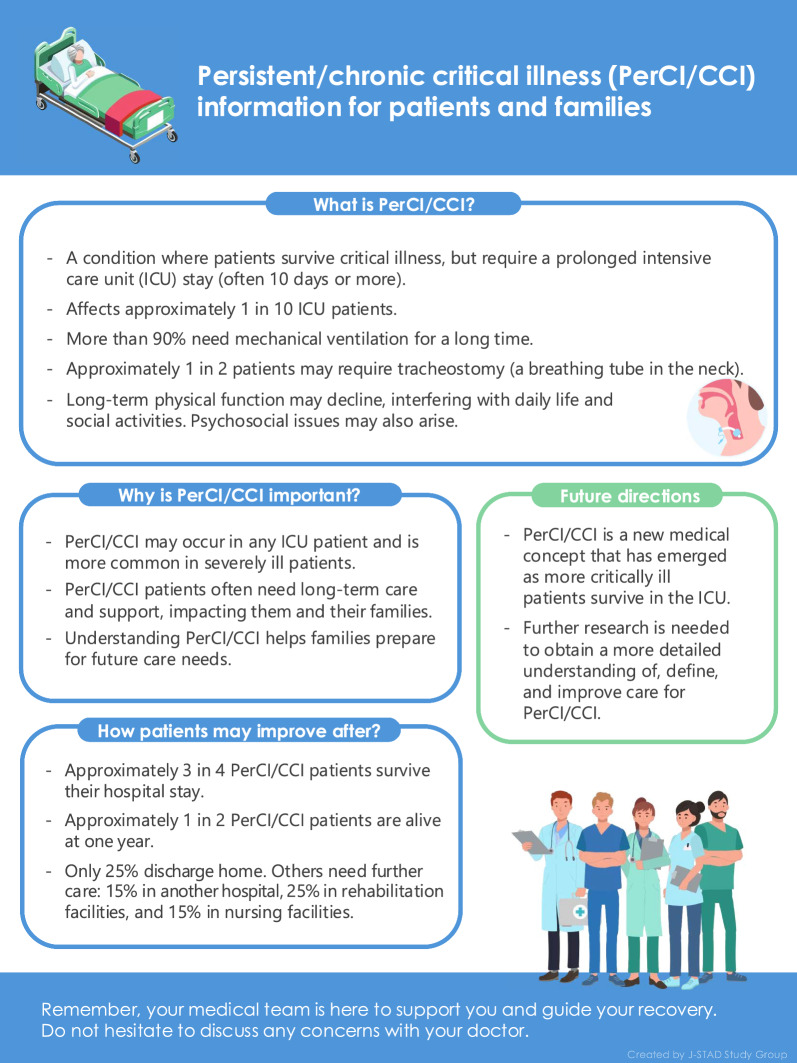
Layperson-friendly infographic of this review for patients with PerCI/CCI and their families. *PerCI* persistent critical illness; *CCI* chronic critical illness; *ICU* intensive care unit

In this review approximately 10% of all ICU patients developed PerCI/CCI and its prevalence was even higher in severe acute illnesses, including sepsis (28%), trauma (24%), and COVID-19 (35%). Figure 4 provides a comprehensive overview of the results obtained in this review. Figure 5 may be also used to improve healthcare providers’ awareness and understanding of these conditions and as well as to facilitate effective communication with patients and their families. We identified higher severity of illness as a risk factor for PerCI/CCI, while previous studies reported that patients with sepsis [119], trauma [120], and COVID-19 [121] had higher severity of illness than all patients admitted to the ICU. Another reason may be severe inflammation and the high prevalence of PIICS in these diseases, which are associated with PerCI/CCI [3]. Recognizing the high-risk disease categories for developing PerCI/CCI, such as sepsis and trauma, can enhance the quality of clinical research on these conditions as well as allow for better information sharing with patients and their families at the time of ICU admission.

A recent systematic review on PIICS identified similar risk factors to those in the present study, including an older age, more severe illness, and increased comorbidities [3]. These findings suggest an overlap with PIICS for PerCI/CCI, emphasizing shared pathophysiological mechanisms. However, the relationship between PIICS and PerCI/CCI remains unclear, raising questions such as whether PIICS is a consequence of ongoing organ dysfunction in PerCI/CCI, whether PIICS is a pathophysiological mechanism of PerCI/CCI, or whether PIICS is an endotype of PerCI/CCI [3]. Therefore, further research is needed to elucidate these complex relationships.

Over the past two decades, the accumulation of knowledge on PerCI/CCI patients and their families has led to the present review results. The results obtained herein indicate that 25% of PerCI/CCI patients died in-hospital and more than 50% of PerCI/CCI survivors required long-term care hospitals or skilled nursing homes, with a very high one-year mortality rate of 45%. These patients commonly had impaired functional capacity in their activities of daily living and often become significantly dependent on others at 12 months after ICU admission. Providing adequate information on the possibility of these impairments in layperson-friendly terms is essential for effective communication with patients and their families and for reaching the correct consensus [122–124].

Identifying patients at risk of PerCI/CCI and clarifying their life and functional prognosis may aid in providing appropriate end-of-life care. Camhi et al. found that only 21% of CCI patients had appointed a healthcare proxy, and 16% had expressed treatment preferences through advance directives [117]. Given the severity and long-term nature of brain dysfunction in patients with CCI [125], difficulties are associated with achieving the goals of care conversions, including the withdrawal of life-supporting treatment. It has also been pointed out that older ICU survivors, including PerCI/CCI patients, may have unmet palliative care needs [22]. While the present study identified risk factors for PerCI/CCI, such as an advanced age, high severity, and the need for organ support, studies on this topic remain scattered and insufficient at present.

### Future perspectives

Future research needs to focus on the standardization of definitions for PerCI/CCI, which will enhance the comparability of studies and contribute to more consistent care pathways. Additionally, more studies are needed to examine the long-term outcomes of patients with PerCI/CCI, including functional outcomes and quality of life. The impact of providing prognostic information to clinicians as well as patients and families with PerCI/CCI, including the results of this scoping review, will also need to be investigated. PerCI/CCI is still a new disease concept and will need to be systematically reviewed again in the future.

### Strengths and limitations

This review’s strength lies in its systematic approach, which enabled the comprehensive identification and analysis of a wide range of original articles. Despite its strengths, this scoping review has some limitations. To make our review more feasible, we were only able to include 5 terms for PerCI/CCI. Therefore, it is possible that terms other than the five listed above or studies that did not use these terms, but examined PerCI/CCI were not included. Furthermore, there was large heterogeneity in the quality, definitions, and populations of the studies reviewed, which may have affected the generalizability of the results obtained. In addition, there may have been a publication bias, with more severe cases or better performing facilities more likely to be reported. Moreover, this review focused on adults because children with PerCI/CCI have significantly different definitions, characteristics, recovery patterns, and treatment approaches [126]. Therefore, the present results cannot be generalized to the pediatric population. Another limitation is the lack of a description of long-COVID in the study on COVID. Further studies are needed to investigate how long-COVID symptoms relate to PerCI/CCI and PICS. Moreover, since this study used a scoping review methodology, the data charting process may be arbitrary by the reviewer.

## Conclusion

This scoping review synthesized a wide range of studies on PerCI/CCI, highlighting the serious impact of PerCI/CCI on patients’ long-term outcomes. The present results underscore the need for consistent terminology with high-quality research to further elucidate risk factors and improve patient care. The results obtained provide important information to be used in discussions with patients and families regarding the definitions, prevalence, risk factors, and outcomes associated with these conditions. However, there is still need for higher quality original research studies using consistent terminology with long term follow-up data.

## Supplementary Information

Supplementary Material 1.

Supplementary Material 2.

Supplementary Material 3.

## Data Availability

No datasets were generated or analysed during the current study.

## Abbreviations

ICU: Intensive care unit
PerCI: Persistent critical illness
CCI: Chronic critical illness
PICS: Post-intensive care syndrome
PIICS: Persistent inflammatory, immunosuppressive, and catabolic syndrome
PRISMA-ScR: Preferred Reporting Items for Systematic Reviews and Meta-Analyses extension for Scoping Reviews
APACHE: Acute physiology and chronic health evaluation
IQR: Interquartile range
MV: Mechanical ventilation
COVID-19: Corona virus disease 2019
CI: Confidence interval
