# Supplementary material for: Definitions, epidemiology, and outcomes of persistent/chronic critical illness: a scoping review for translation to clinical practice
Source: Crit Care. 2024 Dec 28;28:435. doi: 10.1186/s13054-024-05215-4 (PMC11681689; doi:10.1186/s13054-024-05215-4)
Supplement: Supplementary file 1 — Supplementary Material 1. [file 13054_2024_5215_MOESM1_ESM.pdf]

Supplementary Material

Definitions, epidemiology, and outcomes of Persistent/Chronic Critical Illness: A scoping review for translation to clinical practice

Hiroyuki Ohbe, Kasumi Satoh, Takaaki Totoki, Atsushi Tanikawa, Kasumi Shirasaki, Yoshihide Kuribayashi, Miku Tamura, Yudai Takatani, Hiroyasu Ishikura, Kensuke Nakamura; J-STAD (JAPAN Sepsis Treatment and Diagnosis) Study Group

Search strategy for the systematic literature review

MEDLINE (PubMed)

#1	“persistent critical illness”[All Fields] “chronic critical illness”[All Fields] OR “chronically critically ill”[All Fields] OR “chronic critically ill”[All Fields] OR "prolonged critical illness"

Scopus

TITLE-ABS-KEY ("persistent critical illness" OR "chronic critical illness" OR "chronically critically ill" OR "chronic critically ill" OR "prolonged critical illness")

Supplemental Figure 1. Publication year and number of publications stratified by five terminologies for PerCI/CCI.

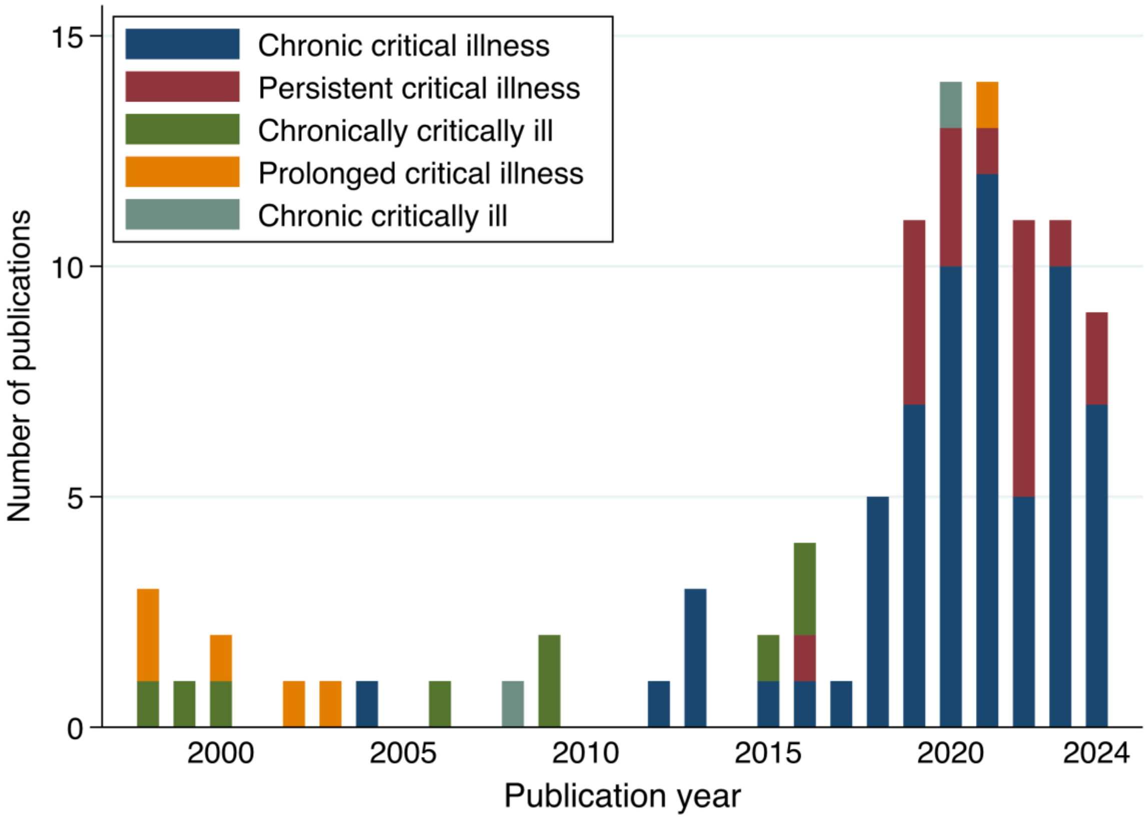

PerCI, persistent critical illness; CCI, chronic critical illness.

Supplemental Figure 2. Meta-analysis for prevalence of “chronic critical illness”.

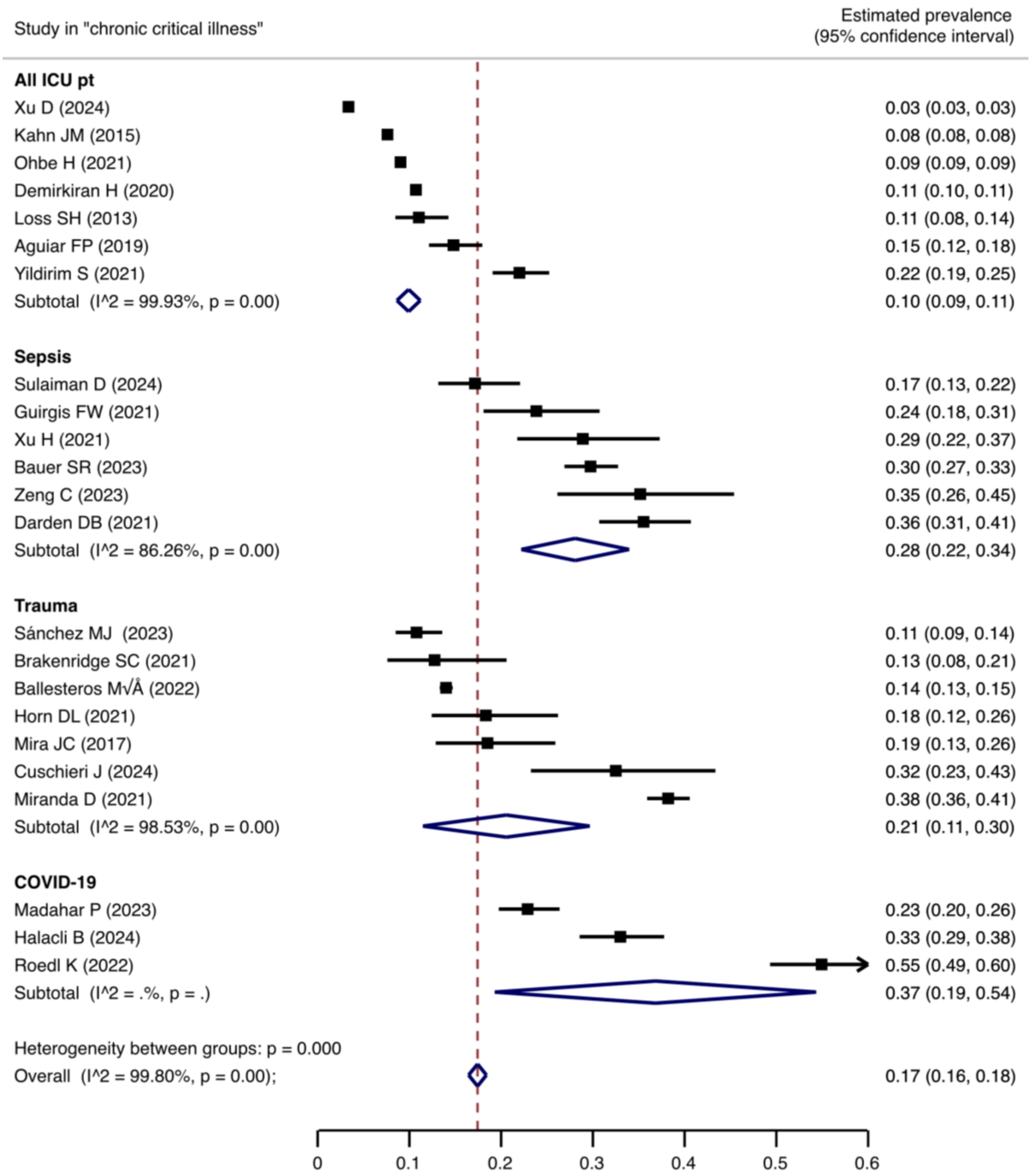

The summary statistics (diamonds) for each stratum and all studies overall are the results of a random effects model. ES, CI, ICU, intensive care unit; COVID-19, corona virus infection disease 2019.

Supplemental Figure 3. Meta-analysis for prevalence of "persistent critical illness".

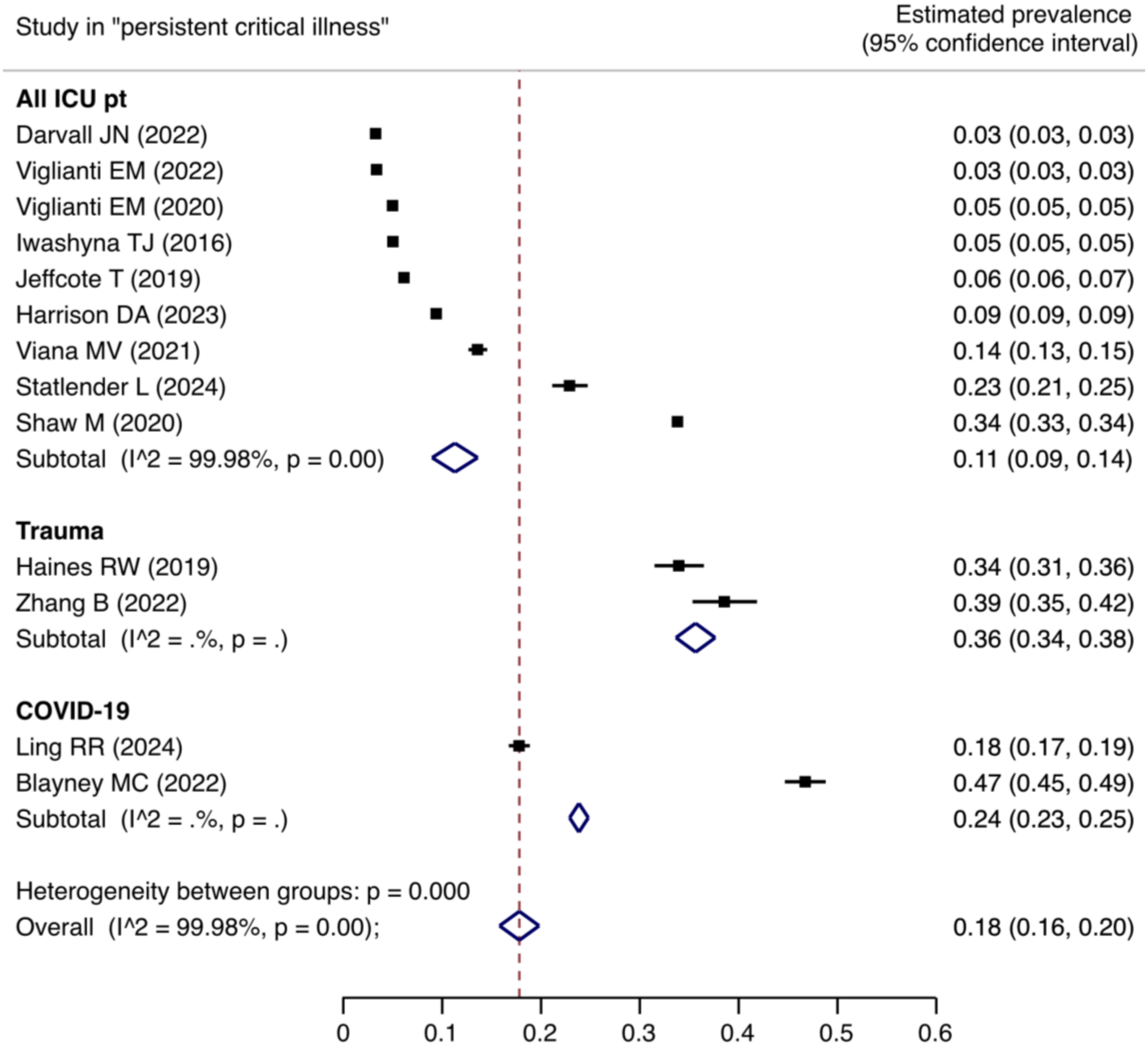

The summary statistics (diamonds) for each stratum and all studies overall are the results of a random effects model. ICU, intensive care unit; COVID-19, corona virus infection disease 2019.

Supplemental Figure 4. Meta-analysis for prevalence of PerCI/CCI stratified by seven continents.

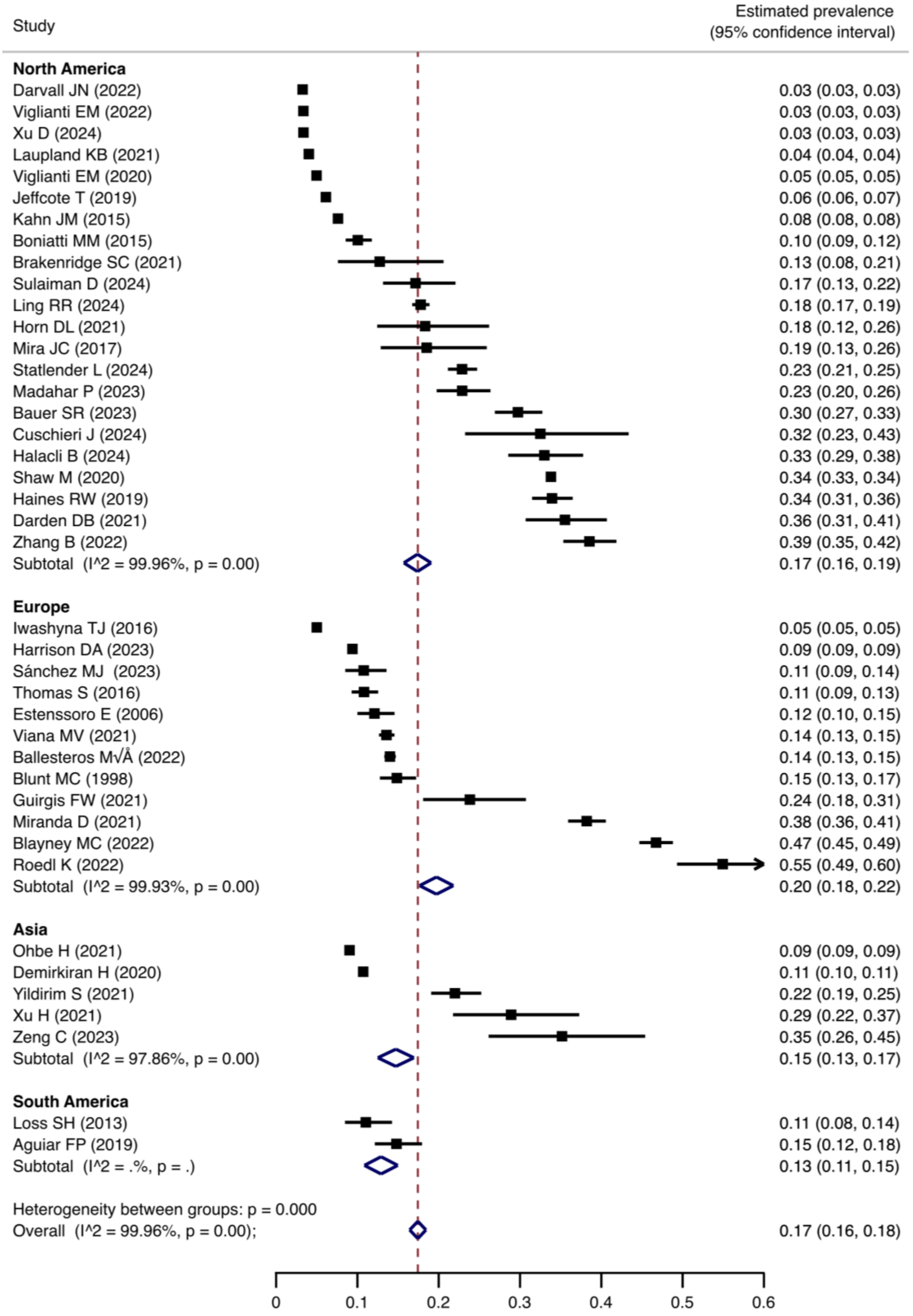

The summary statistics (diamonds) for each stratum and all studies overall are the results of a random effects model. PerCI, persistent critical illness; CCI, chronic critical illness.

Supplemental Figure 5. Meta-analysis for age of PerCI/CCI.

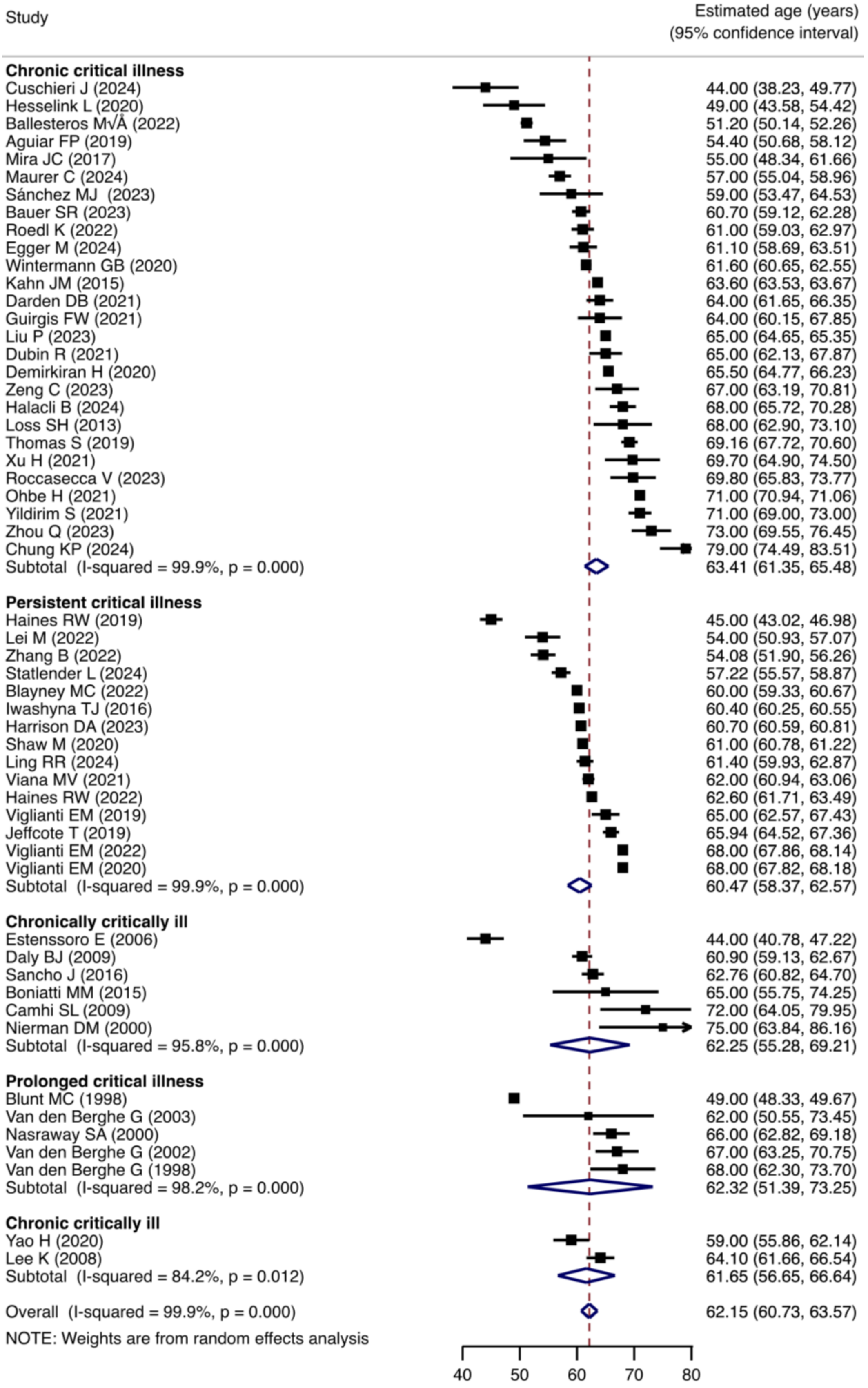

The summary statistics (diamonds) for each stratum and all studies overall are the results of a random effects model.

Supplemental Figure 6. Meta analysis of APACHE Ⅱ score of PerCI/CCI.

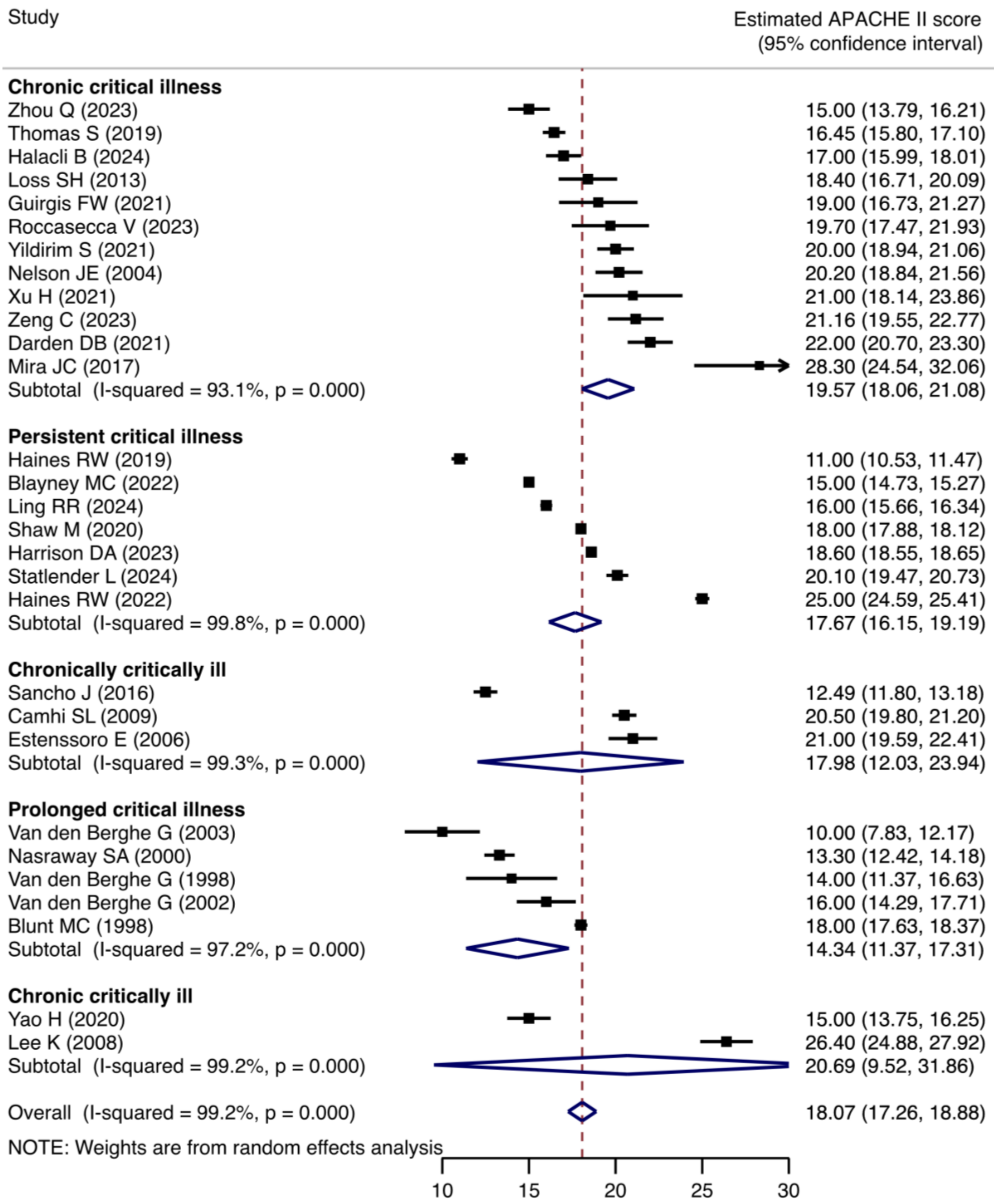

The summary statistics (diamonds) for each stratum and all studies overall are the results of a random effects model. APACHE, acute physiology and chronic health evaluation

Supplemental Figure 7. Meta-analysis for in-hospital mortality of PerCI/CCI stratified by seven continents.

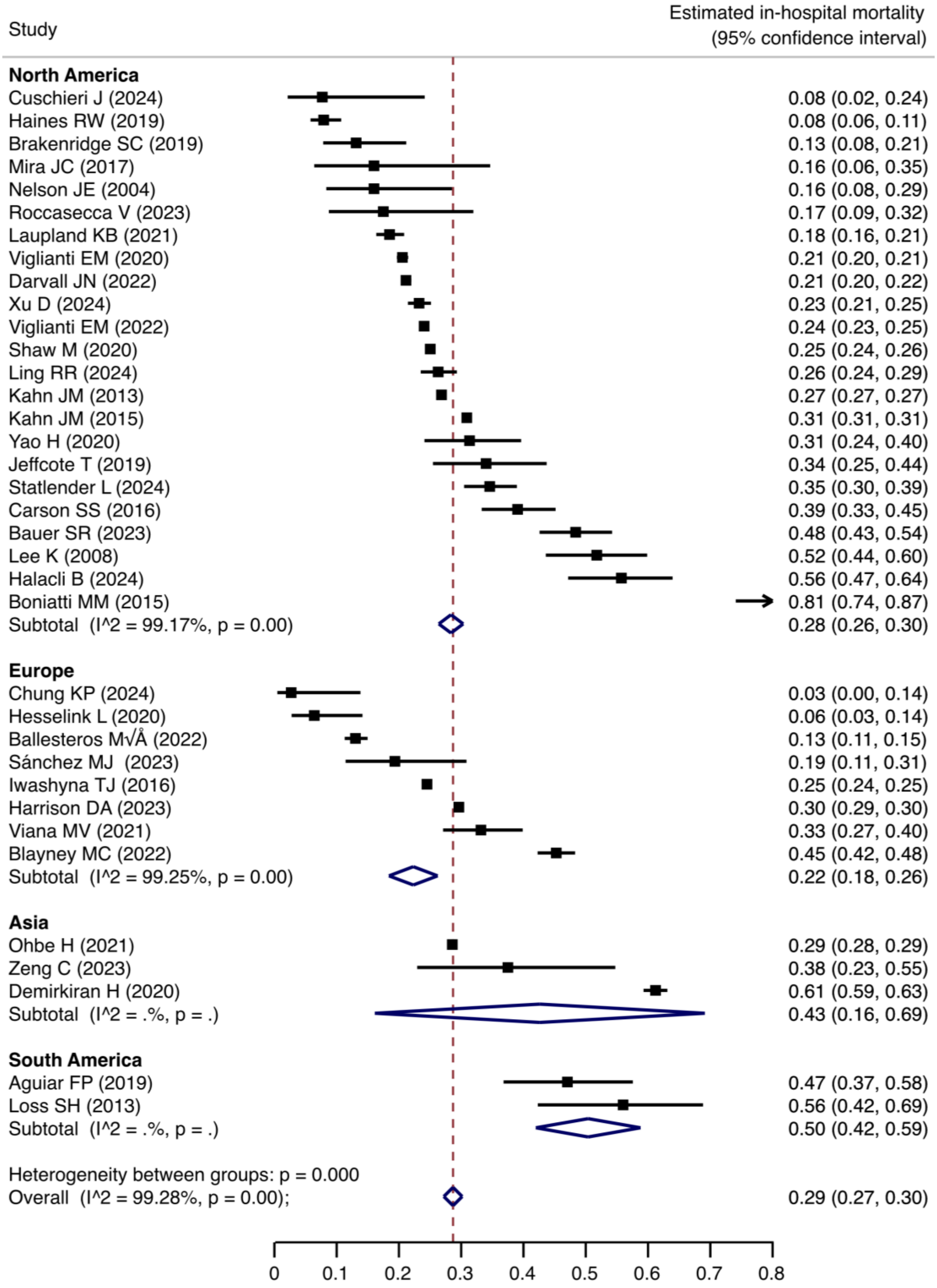

The summary statistics (diamonds) for each stratum and all studies overall are the results of a random effects model. PerCI, persistent critical illness; CCI, chronic critical illness.

Supplemental Figure 8. Meta-analysis for one-year mortality of "chronic critical illness".

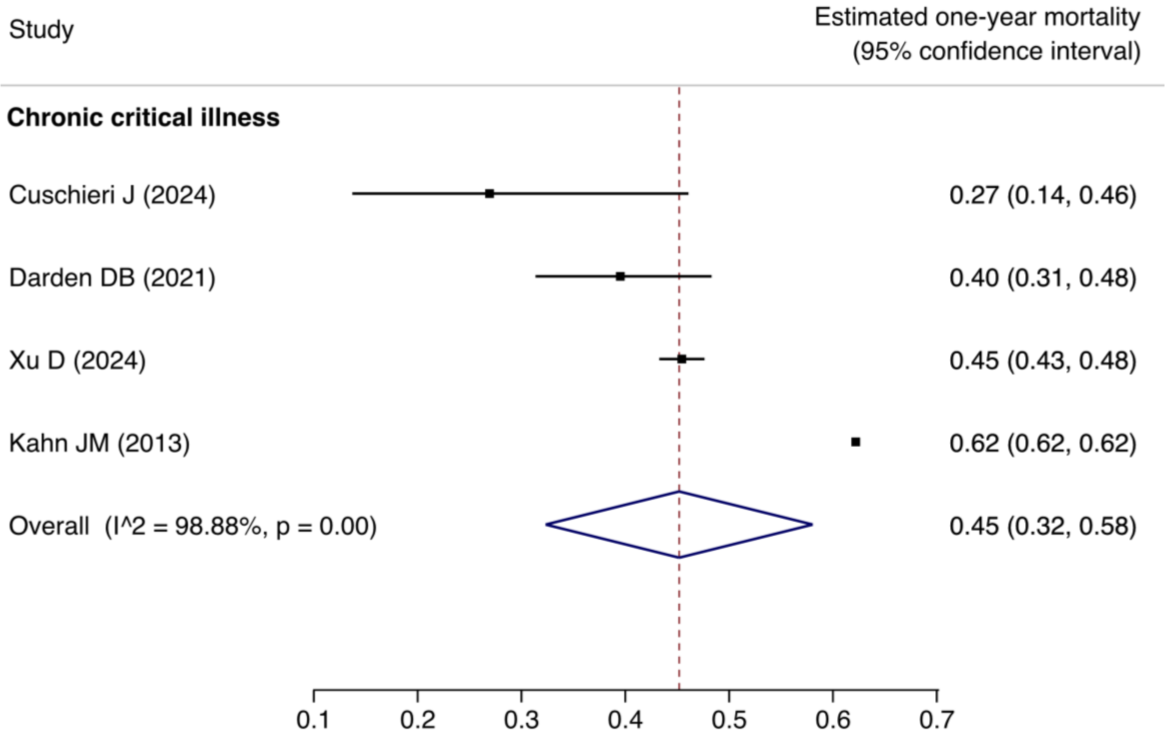

The summary statistics (diamonds) for each stratum and all studies overall are the results of a random effects model.

Supplemental Table 1. Terminologies and definitions of persistent/chronic critical illness

Authors	Year	Country	Types of study	Terminology	Number of PerCI/CCI	PerCI/CCI definition
Nelson JE [20]	2004	United States	Observational	Chronic critical illness	50	Tracheotomy and transfer to the respiratory care unit.
Via MA [21]	2012	United States	RCTs	Chronic critical illness	20	ICU stay ≥5 days and tracheostomy.
Baldwin MR [22]	2013	United States	Observational	Chronic critical illness	38	Tracheostomy after >10 days of MV.
Kahn JM [23]	2013	United States	Observational	Chronic critical illness	234799	ICU stay ≥14 days and MV.
Loss SH [24]	2013	Brazil	Observational	Chronic critical illness	50	ICU stay ≥20 days and MV or hemodynamic support.
Kahn JM [25]	2015	United States	Observational	Chronic critical illness	246151	ICU stay ≥8 days and one of six eligible clinical conditions*.
Carson SS [26]	2016	United States	RCTs	Chronic critical illness	256	MV ≥7 days.
Mira JC [27]	2017	United States	Observational	Chronic critical illness	25	ICU stay ≥14 days with evidence of ongoing organ dysfunction.
Brakenridge SC [28]	2018	United States	Observational	Chronic critical illness	84	ICU stay ≥14 days with persistent organ dysfunction by SOFA.
Davoudi A [29]	2018	United States	Observational	Chronic critical illness	5	ICU stay ≥14 days with persistent organ dysfunction.
Stortz JA [30]	2018	United States	Observational	Chronic critical illness	35	ICU stay ≥14 days with persistent organ dysfunction by SOFA.
Stortz JA [31]	2018	United States	Observational	Chronic critical illness	71	ICU stay ≥14 days with persistent organ dysfunction by SOFA.
Thomas S [32]	2018	England	Observational	Chronic critical illness	150	ICU stay ≥14 days and ICU treatment ≥21 days.
Aguiar FP [33]	2019	Brazil	Observational	Chronic critical illness	85	MV ≥21 days.
Brakenridge SC [34]	2019	United States	Observational	Chronic critical illness	55	ICU stay ≥14 days with persistent organ dysfunction by SOFA.
Brakenridge SC [35]	2019	United States	Observational	Chronic critical illness	99	ICU stay ≥14 days with persistent organ dysfunction by SOFA.
Custodero C [36]	2019	England	Observational	Chronic critical illness	74	ICU stay ≥14 days with persistent organ dysfunction by SOFA.
Gardner AK [37]	2019	United States	Observational	Chronic critical illness	63	ICU stay ≥14 days with persistent organ dysfunction by SOFA.
Thomas S [38]	2019	Sweden	Observational	Chronic critical illness	150	ICU stay >21 days including more than 6 h of MV daily.
Viana MV [39]	2019	Switzerland	Observational	Chronic critical illness	150	ICU stay >14 days.
Bento T [40]	2020	United States	Observational	Chronic critical illness	158	Prolonged MV, tracheostomy, organ failure ≥2 systems, sepsis, wounds, and debilitating factors.
Cox MC [41]	2020	United States	Observational	Chronic critical illness	53	ICU stay ≥14 days with persistent organ dysfunction by SOFA.
Demirkiran H [42]	2020	Turkey	Observational	Chronic critical illness	2493	ICU stay ≥8 days with one of six eligible clinical conditions*.
Hawkins RB [43]	2020	United States	Observational	Chronic critical illness	41	ICU stay ≥14 days with persistent organ dysfunction by SOFA.
Hesselink L [44]	2020	Switzerland	Observational	Chronic critical illness	78	ICU stay ≥14 days.
Ingraham NE [45]	2020	United States	Observational	Chronic critical illness	45	MV ≥7 days.
Mankowski RT [46]	2020	United States	Observational	Chronic critical illness	114	ICU stay ≥14 days with persistent organ dysfunction by SOFA.
Rosenthal MD [47]	2020	United States	Observational	Chronic critical illness	56	ICU stay ≥14 days with persistent organ dysfunction by SOFA.

Stortz JA [48]	2020	England	Observational	Chronic critical illness	108	ICU stay ≥14 days with persistent organ dysfunction by SOFA.
Wintermann GB [49]	2020	England	Observational	Chronic critical illness	267	ICU stay ≥6 days, diagnosis of critical illness polyneuropathy or myopathy, and MV.
Brakenridge SC [50]	2021	United States	Observational	Chronic critical illness	13	ICU stay ≥14 days with persistent organ dysfunction.
Cox MC [51]	2021	Germany	Observational	Chronic critical illness	13	ICU stay ≥14 days with persistent organ dysfunction by SOFA.
Darden DB [52]	2021	Switzerland	Observational	Chronic critical illness	79	ICU stay ≥14 days with persistent organ dysfunction by SOFA.
Darden DB [53]	2021	United States	Observational	Chronic critical illness	124	ICU stay ≥14 days with organ dysfunction.
Dubin R [54]	2021	United States	Observational	Chronic critical illness	50	MV >21 days or tracheostomy.
Guirgis FW [55]	2021	England	Observational	Chronic critical illness	41	ICU stay>14 days with organ dysfunction or ICU≤14 days, but discharged to another hospital or hospice.
Horn DL [56]	2021	United States	Observational	Chronic critical illness	22	ICU stay ≥14 days with persistent organ dysfunction.
Miranda D [57]	2021	England	Observational	Chronic critical illness	640	ICU stay ≥14 days with ongoing organ dysfunction defined by the Modified Marshall Score.
Ohbe H [58]	2021	United States	Observational	Chronic critical illness	216434	ICU stay ≥8 days with one of six eligible clinical conditions*.
Wendlandt B [59]	2021	United States	Observational	Chronic critical illness	256	MV ≥7 days.
Xu H [60]	2021	China	Observational	Chronic critical illness	37	ICU stay ≥14 days with persistent organ dysfunction by SOFA.
Yildirim S [61]	2021	India	Observational	Chronic critical illness	152	ICU stay ≥21 days.
Ballesteros MÅ [62]	2022	England	Observational	Chronic critical illness	1290	MV ≥14 days or tracheostomy.
Madushani RWMA [63]	2022	United States	Observational	Chronic critical illness	50	ICU stay ≥14 days with persistent organ dysfunction by SOFA.
Mankowski RT [64]	2022	United States	Observational	Chronic critical illness	61	ICU stay ≥14 days with persistent organ dysfunction by SOFA.
Roedl K [65]	2022	Switzerland	Observational	Chronic critical illness	167	ICU stay ≥21 days.
Wendlandt B [66]	2022	United States	Observational	Chronic critical illness	224	MV ≥7 days.
Balch JA [67]	2023	England	Observational	Chronic critical illness	132	ICU stay ≥14 days with persistent organ dysfunction by SOFA.
Bauer SR [68]	2023	United States	Observational	Chronic critical illness	279	ICU stay ≥14 days with persistent organ dysfunction by SOFA.
Carmichael ED [69]	2023	United States	Observational	Chronic critical illness	79	ICU stay ≥14 days with persistent organ dysfunction.
Liu P [70]	2023	England	Observational	Chronic critical illness	8145	ICU stay ≥14 days with persistent organ dysfunction by SOFA.
Madahar P [71]	2023	United States	Observational	Chronic critical illness	141	MV >21 days, tracheostomy, and survival to ICU discharge.
Roccasecca V [72]	2023	United States	Observational	Chronic critical illness	40	ICU stay ≥8 days with one of six eligible clinical conditions*.
Sánchez MJ [73]	2023	England	Observational	Chronic critical illness	62	MV ≥14 days or tracheostomy.
Stern K [74]	2023	United States	Observational	Chronic critical illness	520	SOFA score of 3 or higher on ICU day 13, 14, or 15.
Zeng C [75]	2023	China	Observational	Chronic critical illness	32	ICU stay ≥14 days with persistent organ dysfunction by SOFA.
Zhou Q [76]	2023	United States	Observational	Chronic critical illness	71	ICU stay ≥14 days and one of six eligible clinical conditions*.
Chung KP [77]	2024	Switzerland	Observational	Chronic critical illness	37	MV ≥10 days.

Cuschieri J [78]	2024	United States	Observational	Chronic critical illness	26	ICU stay ≥14 days with ongoing organ dysfunction defined by the Modified Marshall Score.
Egger M [79]	2024	England	Observational	Chronic critical illness	97	ICU stay ≥8 days and one of six eligible clinical conditions.
Halacli B [80]	2024	United States	Observational	Chronic critical illness	131	ICU stay ≥14 days with persistent organ dysfunction by SOFA.
Maurer C [81]	2024	Switzerland.	Observational	Chronic critical illness	227	ICU stay ≥7 days.
Sulaiman D [82]	2024	United States	Observational	Chronic critical illness	47	ICU stay ≥14 days with persistent organ dysfunction by SOFA.
Xu D [83]	2024	United States	Observational	Chronic critical illness	2588	ICU stay ≥14 days with persistent organ dysfunction by SOFA.
Iwashyna TJ [84]	2016	England	Observational	Persistent critical illness	51509	ICU stay >10 days
Darvall JN [85]	2019	Netherlands	Observational	Persistent critical illness	245	ICU stay >10 days.
Haines RW [86]	2019	United States	Observational	Persistent critical illness	467	ICU stay ≥10 days.
Jeffcote T [87]	2019	United States	Observational	Persistent critical illness	741	ICU stay >10 days, MV for >24 h, and no limitation on treatment orders on admission.
Viglianti EM [88]	2019	Netherlands	Observational	Persistent critical illness	181	ICU stay ≥10 days.
Shaw M [89]	2020	United States	Observational	Persistent critical illness	24425	ICU stay ≥5 days.
Tseitkin B [90]	2020	Netherlands	Observational	Persistent critical illness	324	ICU stay ≥10 days.
Viglianti EM [91]	2020	United States	Observational	Persistent critical illness	7640	ICU stay >10 days.
Viana MV [92]	2021	England	Observational	Persistent critical illness	680	ICU stay >10 days.
Blayney MC [93]	2022	England	Observational	Persistent critical illness	1045	ICU stay ≥10 days.
Darvall JN [94]	2022	United States	Observational	Persistent critical illness	8814	ICU stay >10 days.
Haines RW [95]	2022	United States	Observational	Persistent critical illness	1021	ICU stay >10 days.
Lei M [96]	2022	Switzerland	Observational	Persistent critical illness	201	ICU stay >10 days.
Viglianti EM [97]	2022	United States	Observational	Persistent critical illness	13184	ICU stay >14 days.
Zhang B [98]	2022	United States	Observational	Persistent critical illness	328	ICU stay >10 days.
Harrison DA [99]	2023	England	Observational	Persistent critical illness	78536	ICU stay >10 days.
Ling RR [100]	2024	United States	Observational	Persistent critical illness	882	ICU stay ≥10 days.
Statlender L [101]	2024	United States	Observational	Persistent critical illness	480	ICU stay >10 days.
Nierman DM [102]	1998	United States	Observational	Chronically critically ill	49	Ventilator-dependent ICU patient with tracheotomy.
Nierman DM [103]	1999	United States	Observational	Chronically critically ill	30	Ventilator-dependent ICU patient with tracheotomy.
Nierman DM [104]	2000	United States	Observational	Chronically critically ill	55	Ventilator-dependent ICU patient with tracheotomy.
Estenssoro E [105]	2006	England	Observational	Chronically critically ill	95	Tracheotomy for MV.
Camhi SL [106]	2009	United States	Observational	Chronically critically ill	203	Patients admitted to the respiratory care unit.
Daly BJ [107]	2009	United States	Observational	Chronically critically ill	334	MV >72 hours and survived during hospitalization.

Boniatti MM [108]	2015	United States	Observational	Chronically critically ill	135	MV ≥21 days.
Sancho J [109]	2016	England	Observational	Chronically critically ill	231	Survived, but not yet recovered to the point of liberation from life-sustaining therapies.
Thomas S [110]	2016	Sweden	Observational	Chronically critically ill	150	ICU stay ≥14 days and ICU treatment ≥21 days.
Blunt MC [111]	1998	England	Observational	Prolonged critical illness	145	ICU stay ≥7 days.
Van den Berghe G [112]	1998	United States	RCTs	Prolonged critical illness	20	ICU stay ≥12 days and required MV.
Nasraway SA [113]	2000	United States	Observational	Prolonged critical illness	97	ICU stay ≥7 days.
Van den Berghe G [114]	2002	England	RCTs	Prolonged critical illness	33	ICU stay >14 days.
Van den Berghe G [115]	2003	United States	RCTs	Prolonged critical illness	22	MV >7 days or patients treated in step-down units.
Laupland KB [116]	2021	United States	Observational	Prolonged critical illness	1157	ICU stay ≥14 days.
Lee K [117]	2008	United States	Observational	Chronic critically ill	141	ICU stay ≥21 days and required MV.
Yao H [118]	2020	Canada	Observational	Chronic critically ill	134	Tracheostomy.

PerCI, persistent critical illness; CCI, chronic critical illness; ICU, intensive care unit; MV, mechanical ventilation.

Supplemental Table 2. Serial studies

Serial groups	Authors	Year	Country	Terminology	Number of PerCI/CCI	Cohort
1	Brakenridge SC [28]	2018	United States	Chronic critical illness	84	University of Florida, Sepsis and Critical Illness Research Center (SCIRC)
1	Davoudi A [29]	2018	United States	Chronic critical illness	5	
1	Stortz JA [30]	2018	United States	Chronic critical illness	35	
1	Stortz JA [31]	2018	United States	Chronic critical illness	71	
1	Brakenridge SC [34]	2019	United States	Chronic critical illness	55	
1	Brakenridge SC [35]	2019	United States	Chronic critical illness	99	
1	Custodero C [36]	2019	England	Chronic critical illness	74	
1	Gardner AK [37]	2019	United States	Chronic critical illness	63	
1	Cox MC [41]	2020	United States	Chronic critical illness	53	
1	Hawkins RB [43]	2020	United States	Chronic critical illness	41	
1	Mankowski RT [46]	2020	United States	Chronic critical illness	114	
1	Rosenthal MD [47]	2020	United States	Chronic critical illness	56	
1	Stortz JA [48]	2020	England	Chronic critical illness	108	
1	Cox MC [51]	2021	Germany	Chronic critical illness	13	
1	Darden DB [52]	2021	Switzerland	Chronic critical illness	79	
1	Darden DB [53]	2021	United States	Chronic critical illness	124	
1	Madushani RWMA [63]	2022	United States	Chronic critical illness	50	
1	Mankowski RT [64]	2022	United States	Chronic critical illness	61	
1	Balch JA [67]	2023	England	Chronic critical illness	132	
1	Carmichael ED [69]	2023	United States	Chronic critical illness	79	
2	Iwashyna TJ [84]	2016	England	Persistent critical illness	51509	Australian and New Zealand Intensive Care Society Adult Patient Database (ANZCIS APD)
2	Darvall JN [85]	2019	Netherlands	Persistent critical illness	245	
2	Tseitkin B [90]	2020	Netherlands	Persistent critical illness	324	
3	Carson SS [26]	2016	United States	Chronic critical illness	256	Randomized Clinical Trial in the United States and their post-hoc analyses
3	Wendlandt B [59]	2021	United States	Chronic critical illness	256	
3	Wendlandt B [66]	2022	United States	Chronic critical illness	224	
4	Nierman DM [102]	1998	United States	Chronically critically ill	49	Mt. Sinai Medical Center, respiratory care unit
4	Nierman DM [103]	1999	United States	Chronically critically ill	30	

4	Nierman DM [104]	2000	United States	Chronically critically ill	55	
5	Thomas S [32]	2018	England	Chronic critical illness	150	General Weakness Syndrome Therapy (GymNAST) study
5	Thomas S [38]	2019	Sweden	Chronic critical illness	150	
5	Thomas S [110]	2016	Sweden	Chronically critically ill	150	
6	Viana MV [39]	2019	Switzerland	Chronic critical illness	150	Lausanne University Hospital, ICU
6	Viana MV [92]	2021	England	Persistent critical illness	680	

Supplemental Table 3. Prevalence and characteristics of included studies

Authors	Prevalence, n (%)	Denominator	Age, years	Male, %	MV, %	Tracheo-stomy, %	Severity at admission
**Chronic critical illness**							
Nelson JE [20]	NA	NA	median 73	52	100	100	APACHE II mean 20.2
Via MA [21]	20/45 (44)	Other	NA	NA	100	100	NA
Baldwin MR [22]	38/228 (17)	Other	NA	NA	NA	NA	NA
Kahn JM [23]	NA	NA	NA	49	100	NA	NA
Loss SH [24]	50/453 (11)	All ICU pts	mean 68	42	50	NA	APACHE II mean 18.4, SOFA mean 4.7
Kahn JM [25]	246,151/3,234,741 (7.6)	All ICU pts	mean 64	55	72	4	NA
Carson SS [26]	NA	NA	mean 59	51	100	54	NA
Mira JC [27]	25/135 (19)	Trauma	mean 55	76	96	NA	APACHE II mean 28.3, ISS mean 34.9
Brakenridge SC [28]	84/173 (49)	Sepsis	NA	NA	NA	NA	NA
Davoudi A [29]	NA	NA	median 63	60	NA	NA	NA
Stortz JA [30]	35/88 (40)	Sepsis	mean 60	66	NA	NA	APACHE II mean 20.9
Stortz JA [31]	71/145 (49)	Sepsis	mean 63	66	NA	NA	APACHE II mean 23.6
Thomas S [32]	NA	NA	mean 69	NA	100	NA	APACHE II median 16
Aguiar FP [33]	85/574 (15)	All ICU pts	mean 54	66	100	34	SAPS III mean 64.6, SOFA mean 7.1
Brakenridge SC [34]	55/157 (35)	Sepsis	median 65	66	NA	NA	APACHE II median 21, SOFA median 10
Brakenridge SC [35]	99/301 (33)	Sepsis	mean 62	65	NA	NA	APACHE II median 22
Custodero C [36]	74/224 (33)	Sepsis	mean 62	62	NA	NA	APACHE II mean 21.4
Gardner AK [37]	63/173 (36)	Sepsis	mean 63	60	NA	NA	APACHE II median 21
Thomas S [38]	NA	NA	mean 70	NA	100	NA	APACHE II median 16
Viana MV [39]	NA	NA	mean 60	77	NA	NA	SAPS II mean 52.9, SOFA median 8
Bento T [40]	158/179 (88)	Other	NA	NA	NA	NA	NA
Cox MC [41]	53/144 (37)	Sepsis	median 66	60	96	NA	APACHE II median 22
Demirkiran H [42]	2493/23272 (10.7)	All ICU pts	mean 66	58.6	95	30.6	NA
Hawkins RB [43]	41/112 (37)	Sepsis	median 64	61	93	NA	APACHE II median 22, SOFA median 10
Hesselink L [44]	NA	NA	median 49	81	100	58	ISS mean 34
Ingraham NE [45]	45/382 (12)	Other	mean 53	67	29	NA	NA
Mankowski RT [46]	114/328 (35)	Sepsis	NA	NA	NA	NA	NA
Rosenthal MD [47]	NA	NA	mean 60	57	NA	NA	APACHE II median 20
Stortz JA [48]	108/316 (34)	Sepsis	NA	NA	NA	NA	NA

Wintermann GB [49]	NA	NA	median 62	72	100	NA	NA
Brakenridge SC [50]	13/102 (13)	Trauma	NA	NA	NA	NA	NA
Cox MC [51]	13/47 (28)	Sepsis	NA	NA	NA	NA	NA
Darden DB [52]	79/118 (67)	Sepsis	mean 62	61	NA	NA	APACHE II median 22, SOFA median 10
Darden DB [53]	124/349 (36)	Sepsis	median 64	64	94	NA	APACHE II median 22
Dubin R [54]	NA	NA	median 65	48	100	100	NA
Guirgis FW [55]	41/172 (24)	Sepsis	median 64	61	88	NA	APACHE II median 19, SOFA median 7
Horn DL [56]	22/120 (18)	Trauma	NA	NA	NA	NA	NA
Miranda D [57]	640/1,675 (38)	Trauma	NA	NA	NA	NA	NA
Ohbe H [58]	216434/2,395,016 (9.0)	All ICU pts	median 71	61	74	24	NA
Wendlandt B [59]	NA	NA	mean 59	51	100	54	NA
Xu H [60]	37/128 (29)	Sepsis	mean 70	62	NA	NA	APACHE II median 21, SOFA median 9
Yildirim S [61]	152/691 (22)	All ICU pts	median 71	65	NA	51	APACHE II median 20
Ballesteros MÅ [62]	1290/9,213 (14)	Trauma	mean 51	80	100	78	ISS mean 26.3
Madushani RWMA [63]	50/157 (32)	Sepsis	NA	NA	NA	NA	NA
Mankowski RT [64]	61/145 (42)	Sepsis	median 72	62	92	NA	APACHE II median 23
Roedl K [65]	167/304 (55)	COVID	median 61	70	86	51	SAPS II mean 39.5, SOFA mean 10
Wendlandt B [66]	NA	NA	mean 59	51	100	54	NA
Balch JA [67]	132/522 (25)	Other	NA	NA	NA	NA	NA
Bauer SR [68]	279/938 (30)	Sepsis	mean 60	54	65	NA	APACHE III median 97.2, SOFA median 12.5
Carmichael ED [69]	79/205 (39)	Sepsis	mean 62	61	NA	NA	NA
Liu P [70]	NA	NA	median 65	58	NA	NA	SOFA mean 6.1
Madahar P [71]	141/616 (23)	COVID	NA	62	NA	NA	NA
Roccasecca V [72]	NA	NA	mean 70	80	100	NA	APACHE II mean 19.7, SAPS III mean 57.2, SOFA mean 6.4
Sánchez MJ [73]	62/575 (11)	Trauma	median 59	84	100	NA	ISS mean 17
Stern K [74]	520/3194 (16)	Other	NA	NA	NA	NA	NA
Zeng C [75]	32/91 (35)	Sepsis	mean 67	68.8	84.4	NA	APACHE II mean 21.16
Zhou Q [76]	71/168 (43)	Other	median 73	66	NA	NA	APACHE II median 15, SOFA median 8
Chung KP [77]	NA	NA	mean 79	54	100	NA	NA
Cuschieri J [78]	26/80 (33)	Trauma	mean 44	81	96	NA	ISS mean 32.4
Egger M [79]	NA	NA	mean 61	69	100	75	NA
Halacli B [80]	131/397 (33)	COVID	median 68	63	76	14	APACHE II median 17, SOFA median 5
Maurer C [81]	NA	NA	mean 57	60.4	NA	NA	NA

Sulaiman D [82]	47/274 (17)	Sepsis	NA	NA	NA	NA	NA
Xu D [83]	2588/76943 (3.4)	All ICU pts	NA	59.3	96	NA	NA
**Persistent critical illness**							
Iwashyna TJ [84]	51,509/1,028,235 (5.0)	All ICU pts	mean 60	62	NA	NA	APACHE III mean 71.7
Darvall JN [85]	245/3,874 (6.3)	All ICU pts	mean 60	64	93	NA	APACHE III mean 82.7
Haines RW [86]	467/1,376 (33)	Trauma	median 45	81	NA	NA	APACHE II median 11, SAPS II median 35
Jeffcote T [87]	741/12,096 (6.1)	All ICU pts	median 66	62	100	NA	APACHE III median 69
Viglianti EM [88]	181/628 (29)	Other	median 65	63	NA	NA	SAPS II mean 29.4
Shaw M [89]	24425/72253 (34)	All ICU pts	median 62	58	NA	NA	APACHE II median 18
Tseitkin B [90]	324/5,402 (6.0)	All ICU pts	median 62	62	97	36	APACHE III median 70
Viglianti EM [91]	7,640/153,512 (5.0)	All ICU pts	median 68	97	NA	NA	NA
Viana MV [92]	680/5,008 (14)	All ICU pts	median 62	76	NA	NA	SAPS II median 45
Blayney MC [93]	1,045/2,236 (47)	COVID	median 60	69	57	NA	APACHE II median 15
Darvall JN [94]	8,814/269,785 (3.3)	All ICU pts	NA	53	NA	NA	NA
Haines RW [95]	NA	NA	mean 63	60	NA	NA	APACHE II median 25, SOFA median 11
Lei M [96]	201/1,257 (16)	Other	median 54	39	NA	NA	SAPS II median 35.0, SOFA median 5
Viglianti EM [97]	13,184/392,731 (3.4)	All ICU pts	median 68	97	NA	18	NA
Zhang B [98]	328/851 (39)	Trauma	mean 54	68	98	NA	SAPS II median 36, SOFA median 5
Harrison DA [99]	78,536/835,946 (9.4)	All ICU pts	mean 61	18	NA	NA	APACHE II mean 18.6
Ling RR [100]	882/4,961 (18)	COVID	median 61	66	79	18	APACHE II median 16
Statlender L [101]	480/2,098 (23)	All ICU pts	mean 57	68	NA	NA	APACHE II mean 20.1, SOFA median 8
**Chronically critically ill**							
Nierman DM [102]	NA	NA	median 73	57	100	100	NA
Nierman DM [103]	NA	NA	median 73	100	100	100	NA
Nierman DM [104]	NA	NA	median 75	55	100	100	NA
Estenssoro E [105]	95/785 (12)	All ICU pts	mean 44	53	100	100	APACHE II mean 21, SOFA mean 7
Camhi SL [106]	NA	NA	median 72	58	100	100	APACHE II mean 20.5
Daly BJ [107]	334/1,041 (32)	Other	median 62	44	100	NA	NA
Boniatti MM [108]	135/1,343 (10)	All ICU pts	median 65	57	NA	84	NA
Sancho J [109]	231/4,609 (5.0)	Other	mean 63	65	68	NA	APACHE II mean 12.5
Thomas S [110]	150/1,387 (11)	All ICU pts	median 71	70	NA	NA	APACHE II median 16
**Prolonged critical illness**							
Blunt MC [111]	145/976 (15)	All ICU pts	mean 49	58	NA	NA	APACHE II mean 18

Van den Berghe G [112]	NA	NA	mean 68	65	NA	NA	APACHE II mean 12
Nasraway SA [113]	NA	NA	mean 66	55	73	NA	APACHE II mean 13
Van den Berghe G [114]	NA	NA	mean 67	100	100	NA	APACHE II mean 16
Van den Berghe G [115]	NA	NA	median 62	54	NA	NA	APACHE II median 10
Laupland KB [116]	1,157/28,742 (4.0)	All ICU pts	median 62	61	NA	NA	NA
**Chronic critically ill**							
Lee K [117]	NA	NA	mean 64	65	100	70	APACHE II mean 26.4, SOFA mean 9.7
Yao H [118]	NA	NA	median 59	53	100	100	APACHE II median 15, SOFA median 6

MV, mechanical ventilation; NA, not applicable; APACHE, acute physiology and chronic health evaluation; ICU, intensive care unit; SOFA, sequential organ failure assessment; ISS, injury severity score; SAPS, simplified acute physiology score; COVID-19, corona virus infection disease 2019.

Supplemental Table 4. Risk factors in included studies

Authors	Terminology	Risk factor
Loss SH [24]	Chronic critical illness	MV, sepsis, GCS <15, inadequate calorie intake, and higher body mass index.
Kahn JM [25]	Chronic critical illness	Older age.
Mira JC [27]	Chronic critical illness	Age ≥55 years, systolic hypotension ≤70 mmHg, transfusion ≥5 units packed red blood cells within 24 hours, and Denver MOF score at 72 hours.
Stortz JA [31]	Chronic critical illness	Delayed onset sepsis (>48 hours after admission), interfacility transfer, vasopressor-dependent septic shock, and SOFA score ≥5 at 72 hours.
Aguiar FP [33]	Chronic critical illness	Neurological diagnosis at hospital admission, pressure ulcers, chronic renal failure, and muscle weakness.
Brakenridge SC [34]	Chronic critical illness	Elevated GLP-1 at 24 hours.
Brakenridge SC [35]	Chronic critical illness	Older age, greater comorbidities, and severe and persistent organ dysfunction.
Cox MC [41]	Chronic critical illness	Higher Charlson Comorbidity Index, early MOF by the Denver score, septic shock, and open abdomen management.
Ingraham NE [45]	Chronic critical illness	Previous liver transplant, acute renal failure, frailty, a lower albumin level, a higher INR, the need for MV, higher systolic pulmonary artery pressure, and lower MELD score.
Mankowski RT [46]	Chronic critical illness	Older age.
Cox MC [51]	Chronic critical illness	Baseline sarcopenia.
Guirgis FW [55]	Chronic critical illness	ApoA-I, LDL-C, MV, vasopressor use, and Charlson Comorbidity Score.
Miranda D [57]	Chronic critical illness	Older age, body mass index, shock, INR ≥1.5, hypothermia, and higher ISS.
Ohbe H [58]	Chronic critical illness	Older age.
Xu H [60]	Chronic critical illness	SIRT2 expression.
Yildirim S [61]	Chronic critical illness	Older age, higher APACHE-2, and vasopressor use.
Ballesteros MÅ [62]	Chronic critical illness	Older age, injury severity score, head injury, infectious complications, and development of MOF.
Mankowski RT [64]	Chronic critical illness	Older age.
Roedl K [65]	Chronic critical illness	ARDS and referral to another ICU.
Sánchez MJ [73]	Chronic critical illness	Surgical intervention in the first 24 hours, age >55 years, ISS score, GCS score, and multiple organ failure.
Zeng C [75]	Chronic critical illness	D7-sPD-L1 and APACHE II scores.
Cuschieri J [78]	Chronic critical illness	Higher ISS score.
Halacli B [80]	Chronic critical illness	Invasive MV and a P/F ratio <150.
Viglianti EM [91]	Persistent critical illness	Hospital-level characteristics.
Blayney MC [93]	Persistent critical illness	Older age, more comorbidities, acute physiology score, and organ support.

Darvall JN [94]	Persistent critical illness	Frailty.
Haines RW [95]	Persistent critical illness	Increased urea-to-creatinine ratio.
Lei M [96]	Persistent critical illness	Dynamic changes in lymphocytes.
Zhang B [98]	Persistent critical illness	Monocyte-to-lymphocyte ratio, ICU type, sepsis, acute kidney injury, MV use, vasopressor therapy, renal replacement therapy, mean systolic blood pressure within the first 24 hours, mean blood glucose level within the first 24 hours, and initial albumin level
Thomas S [110]	Chronically critically ill	Older age, longer MV duration, and lower FSS-ICU scale.

MOF, multiple organ failure; SOFA, sequential organ failure assessment; INR, prothrombin time-international normalized ratio; MELD, model for end-stage liver disease; APACHE, acute physiology and chronic health evaluation; ARDS, acute respiratory distress syndrome; ICU, intensive care unit; ISS, injury severity score; GCS, Glasgow coma scale; FSS, functional status score.

Supplemental Table 5. Outcomes of included studies

Authors	In-hospital mortality, n (%)	One-year mortality, n (%)	Length of ICU, days	Length of hospital, days	Length of MV, days
**Chronic critical illness**					
Nelson JE [20]	8/50 (16.0)	NA	15 (1, 77)	NA	NA
Via MA [21]	NA	NA	NA	NA	NA
Baldwin MR [22]	NA	NA	NA	NA	NA
Kahn JM [23]	63025/234799 (26.8)	146020/234799 (62.2)	NA	NA	NA
Loss SH [24]	28/50 (56.0)	NA	NA	87 ± 77	NA
Kahn JM [25]	76060/246151 (30.9)	NA	18 ± 15	NA	NA
Carson SS [26]	100/256 (39.1)	NA	NA	NA	20 (15, 25)
Mira JC [27]	4/25 (16.0)	NA	27 ± 10	NA	NA
Brakenridge SC [28]	NA	NA	NA	NA	NA
Davoudi A [29]	NA	NA	NA	NA	NA
Stortz JA [30]	4/35 (11.4)	NA	21 (17, 37)	NA	NA
Stortz JA [31]	11/71 (15.5)	NA	22 (16, 34)	NA	16 (8, 23)
Thomas S [32]	NA	NA	NA	NA	NA
Aguiar FP [33]	40/85 (47.1)	NA	29 ± 20	NA	NA
Brakenridge SC [34]	8/55 (14.5)	NA	20 (15, 29)	NA	7 (3, 15)
Brakenridge SC [35]	13/99 (13.1)	41/99 (41.4)	21 (15, 29)	NA	NA
Custodero C [36]	NA	NA	21 (16, 37)	NA	NA
Gardner AK [37]	8/63 (12.7)	28/63 (44.4)	21 (15, 39)	31 (21, 47)	NA
Thomas S [38]	NA	NA	NA	NA	52 (11, 95)
Viana MV [39]	NA	NA	31 (23, 46)	NA	16 (10, 22)
Bento T [40]	NA	NA	NA	NA	NA
Cox MC [41]	NA	22/53 (41.5)	21 (16, 29)	NA	NA
Demirkiran H [42]	1526/2493 (61.2)	NA	21 (13,37)	23 (14, 41)	17(10, 33)
Hawkins RB [43]	NA	18/41 (43.9)	21 (16, 29)	NA	6 (3, 15)
Hesselink L [44]	5/78 (6.4)	NA	20 (16, 29)	43 (35, 63)	20 (15, 27)
Ingraham NE [45]	NA	NA	NA	NA	NA
Mankowski RT [46]	NA	NA	NA	NA	NA
Rosenthal MD [47]	NA	18/56 (32.1)	21 (17, 34)	NA	NA
Stortz JA [48]	NA	NA	NA	NA	NA

Wintermann GB [49]	NA	NA	73 (52, 105)	NA	53 (33, 77)
Brakenridge SC [50]	NA	NA	NA	NA	NA
Cox MC [51]	NA	NA	NA	NA	NA
Darden DB [52]	6/79 (7.6)	33/79 (41.8)	24 (18, 39)	NA	NA
Darden DB [53]	NA	49/124 (39.5)	NA	NA	NA
Dubin R [54]	NA	NA	NA	NA	NA
Guirgis FW [55]	NA	NA	19 (15, 25)	NA	36 ± 88
Horn DL [56]	NA	NA	NA	NA	NA
Miranda D [57]	NA	NA	NA	NA	NA
Ohbe H [58]	61841/216434 (28.6)	NA	13 (10, 14)	47 (28, 78)	NA
Wendlandt B [59]	94/224 (42.0)	NA	NA	NA	20 (15, 25)
Xu H [60]	NA	NA	20 ± 5.3	NA	NA
Yildirim S [61]	NA	NA	31 (25, 48)	31 (25, 48)	NA
Ballesteros MÅ [62]	168/1290 (13.0)	NA	30 ± 20	NA	NA
Madushani RWMA [63]	NA	NA	NA	NA	NA
Mankowski RT [64]	NA	NA	20 (15, 25)	NA	NA
Roedl K [65]	NA	NA	33 (23, 50)	NA	NA
Wendlandt B [66]	94/224 (42.0)	NA	NA	NA	20 (15, 25)
Balch JA [67]	NA	NA	NA	NA	NA
Bauer SR [68]	135/279 (48.4)	NA	NA	NA	NA
Carmichael ED [69]	NA	NA	24 (18, 39)	NA	NA
Liu P [70]	NA	NA	21 (17, 27)	NA	NA
Madahar P [71]	NA	NA	NA	NA	NA
Roccasecca V [72]	7/40 (17.5)	NA	14 (10, 27)	NA	7 (3, 19)
Sánchez MJ [73]	12/62 (19.4)	NA	23 (18, 30)	44 (30, 53)	NA
Stern K [74]	NA	NA	NA	NA	NA
Zeng C [75]	12/32 (37.5)	NA	20 ± 7.1	NA	NA
Zhou Q [76]	NA	NA	24 (18, 57)	NA	NA
Chung KP [77]	1/37 (2.7)	NA	NA	NA	NA
Cuschieri J [78]	2/26 (7.7)	7/26 (26.9)	28 ± 12	NA	NA
Egger M [79]	NA	NA	52 (36, 71)	NA	39 (22, 55)
Halacli B [80]	73/131 (55.7)	NA	24 (18, 33)	NA	18 (11, 27)
Maurer C [81]	NA	NA	16 ± 14	NA	9 ± 6.7
Sulaiman D [82]	NA	NA	NA	NA	NA

Xu D [83]	464/1996 (23.2)	907/1996 (45.4)	NA	NA	NA
**Persistent critical illness**					
Iwashyna TJ [84]	12625/51509 (24.5)	NA	20 ± 15	NA	NA
Darvall JN [85]	23/72 (31.9)	NA	15 (12, 20)	NA	NA
Haines RW [86]	37/467 (7.9)	NA	17 (13, 23)	NA	NA
Jeffcote T [87]	34/100 (34.0)	NA	15 (12, 21)	32 (22, 49)	NA
Viglianti EM [88]	NA	NA	6 (3, 11)	NA	NA
Shaw M [89]	6115/24425 (25.0)	NA	4 (2, 7)	NA	NA
Tseitkin B [90]	88/300 (29.3)	NA	16 (12, 23)	NA	19 (9, 43)
Viglianti EM [91]	1571/7640 (20.6)	NA	15 (12, 20)	NA	NA
Viana MV [92]	68/205 (33.2)	NA	28 (22, 42)	NA	16 (10, 22)
Blayney MC [93]	473/1045 (45.3)	NA	19 (13, 30)	28 ± 19	NA
Darvall JN [94]	1863/8814 (21.1)	NA	NA	NA	NA
Haines RW [95]	NA	NA	11 (6, 28)	NA	NA
Lei M [96]	NA	NA	NA	NA	NA
Viglianti EM [97]	3171/13184 (24.1)	NA	25 (18, 37)	NA	NA
Zhang B [98]	NA	NA	17 (13, 25)	NA	NA
Harrison DA [99]	23267/78536 (29.6)	NA	17 (13, 25)	NA	NA
Ling RR [100]	232/882 (26.3)	NA	17 (13, 25)	25 (19, 40)	NA
Statlender L [101]	166/480 (34.6)	NA	18 (14, 24)	NA	NA
**Chronically critically ill**					
Nierman DM [102]	NA	NA	20 (9,177)	NA	NA
Nierman DM [103]	NA	NA	NA	NA	NA
Nierman DM [104]	NA	NA	16 (1,177)	NA	NA
Estenssoro E [105]	NA	NA	39 (29, 55)	NA	33 (24, 50)
Camhi SL [106]	NA	NA	16 (11, 22)	NA	40 (28, 51)
Daly BJ [107]	NA	NA	13 (16, 19)	NA	8 (11, 13)
Boniatti MM [108]	110/135 (81.5)	NA	36 (28, 49)	NA	33 (26, 46)
Sancho J [109]	NA	NA	45 ± 23	NA	37 ± 24
Thomas S [110]	NA	NA	NA	NA	53 (38, 84)
**Prolonged critical illness**					
Blunt MC [111]	NA	NA	14 (9, 27)	NA	NA
Van den Berghe G [112]	NA	NA	NA	NA	NA

Nasraway SA [113]	NA	NA	39 (7, 276)	NA	33 (0, 251)
Van den Berghe G [114]	NA	NA	NA	NA	NA
Van den Berghe G [115]	NA	NA	18 (13, 30)	NA	NA
Laupland KB [116]	214/1157 (18.5)	264/1157 (22.8)	NA	NA	NA
**Chronic critically ill**					
Lee K [117]	73/141 (51.8)	NA	43 ± 36	84 ± 101	NA
Yao H [118]	42/134 (31.3)	NA	28 (19, 47)	68 (39, 108)	NA

ICU, intensive care unit; MV, mechanical ventilation

Supplemental Table 6. Discharge destinations in included studies

		Discharge destinations					
Authors	Terminology	In-hospital death	Home	Another hospital	Rehabilitation facilities	Nursing facilities	Hospice
Kahn JM [25]	Chronic critical illness	31%	21%	4%	3%	39%	2%
Mira JC [27]	Chronic critical illness	16%	24%	12%	20%	28%	NA
Stortz JA [31]	Chronic critical illness	16%	19%	17%	32%	16%	6%
Hesselink L [44]	Chronic critical illness	6%	35%	10%	35%	14%	NA
Rosenthal MD [47]	Chronic critical illness	NA	11%	16%	46%	18%	9%
Ohbe H [58]	Chronic critical illness	29%	30%	40%	NA	1%	NA
Darvall JN [85]	Persistent critical illness	32%	18%	31%	38%	NA	NA
Jeffcote T [87]	Persistent critical illness	34%	47%	8%	11%	NA	NA
Ling RR [100]	Persistent critical illness	26%	43%	NA	12%	1%	NA

Supplemental Table 7. Functional outcomes in included studies

Authors	Terminology	Functional outcomes
Mira JC [27]	Chronic critical illness	SF-36 at 4 months, mean 42.7
Gardner AK [37]	Chronic critical illness	EQ-5D-3L Health Questionnaire at 12 months Utility Index, mean 0.37, Descriptive System Score, mean 10, VAS, mean 49, Short Physical Performance Total Balance Test, mean 1.03, Hand Grip Strength Measurement, mean 21, Zubrod score, mean 3.4.
Brakenridge SC [35]	Chronic critical illness	SPPB scores at 12 months, mean 3.1, Zubrod scores at 12 months, mean 3.5
Rosenthal MD [47]	Chronic critical illness	Zubrod score at 12 months, mean 3.2, SPPB score, mean 5.0, EQ-5D at 12 months, mean 9, EQ-5D utility at 12 months, mean 0.55.
Cox MC [41]	Chronic critical illness	Zubrod score at 12 months, mean 3.5.
Darden DB [52]	Chronic critical illness	Zubrod at 12 months, median 4.
Mankowski RT [64]	Chronic critical illness	Zubrod score at 12 months, mean 3.5.
Carmichael ED [69]	Chronic critical illness	Gait speed at 12 months, mean 2.1, SPPB scores at 12 months, mean 5.3, Zubrod scores at 12 months, mean 3.4.
Maurer C [81]	Chronic critical illness	EQ-VAS scores at 12 months, mean 73.2, EQ Index at 12 months, mean 0.86.

SF-36, Short-Form 36-Item Health Survey; EQ-5D, EuroQol five-dimensions; VAS, visual analogue scale; SPPB, Short Physical Performance Battery
