# Supplementary material for: Definitions, epidemiology, and outcomes of persistent/chronic critical illness: a scoping review for translation to clinical practice
Source: Crit Care. 2024 Dec 28;28:435. doi: 10.1186/s13054-024-05215-4 (PMC11681689; doi:10.1186/s13054-024-05215-4)
Supplement: Supplementary file 3 — Supplementary Material 3. [file 13054_2024_5215_MOESM3_ESM.pdf]

UMIN-CTR Clinical Trial

UMIN-CTR Home | FAQ | Inquiry

Unique ID issued by UMIN	UMIN000054545
Receipt number	R000062304
Scientific Title	Definitions, epidemiology, and outcomes of Persistent/Chronic Critical Illness: A scoping review for tra
Date of disclosure of the study information	2024/06/02
Last modified on	2024/11/20 02:26:48

* This page includes information on clinical trials registered in UMIN clinical trial registed system.
* We don't aim to advertise certain products or treatments

Basic information

Public title

Persistent/Chronic Critical Illness: a translational scoping review for clinical practice

Acronym

Definitions, epidemiology, and outcomes of Persistent/Chronic Critical Illness: A scoping review for translation to clinical practice

Scientific Title

Definitions, epidemiology, and outcomes of Persistent/Chronic Critical Illness: A scoping review for translation to clinical practice

Scientific Title:Acronym

Definitions, epidemiology, and outcomes of Persistent/Chronic Critical Illness: A scoping review for translation to clinical practice

Region

Japan

Condition

Condition

Persistent critical illness or Chronic critical illness

Classification by specialty

Intensive care medicine

Classification by malignancy

Others

Genomic information

NO

Objectives

Narrative objectives1

We will review original articles on Persistent/Chronic critical illness and organize the evidence on definition, prevalence, risk factors, quality of life.

Basic objectives2

Others

Basic objectives -Others

We will add clinical perspectives to this review evidence and prepare materials that will contribute to the provision of information to critical illness and their families.

Trial characteristics_1

Others

Trial characteristics_2

Others

Developmental phase

Not applicable

Assessment

Primary outcomes

Definition, prevalence, risk factors, prognosis, cost, social burden, and quality of life for persistent/Chronic critical illness.

Key secondary outcomes

Base

Study type

Others,meta-analysis etc

Study design

Basic design

Randomization

Randomization unit

Blinding

Control

Stratification

Dynamic allocation

Institution consideration

Blocking

Concealment

Intervention

No. of arms

Purpose of intervention

Type of intervention

Interventions/Control_1

Interventions/Control_2

Interventions/Control_3

Interventions/Control_4

Interventions/Control_5

Interventions/Control_6

Interventions/Control_7

Interventions/Control_8

Interventions/Control_9

Interventions/Control_10

Eligibility

Age-lower limit

Not applicable

Age-upper limit

Not applicable

Gender

Male and Female

Key inclusion criteria

Include original articles that define patient populations, covariates, and outcomes using the terminology 'persistent critical illness', 'ch
critically ill', 'chronically critically ill', or 'prolonged critical illness'.

Key exclusion criteria

Exclude unpublished studies, preprints, conference abstracts without subsequent study publication, studies not in English, review ar
animal studies, or studies for children.

Target sample size

Research contact person

Name of lead principal investigator

1st name Hiroyuki
Middle name
Last name Ohbe

Organization

Tohoku University Hospital

Division name

Department of Emergency and Critical Care Medicine

Zip code

980-8574

Address

1-1 Seiryo-machi, Aoba-ku, Sendai

TEL

02-2717-7489

Email

hohbey@gmail.com

Public contact

Name of contact person

1st name Hiroyuki
Middle name
Last name Ohbe

Organization

Tohoku University Hospital

Division name

Department of Emergency and Critical Care Medicine

Zip code

980-8574

Address

1-1 Seiryo-machi, Aoba-ku, Sendai

TEL

02-2717-7489

Homepage URL

Email

hohbey@gmail.com

Sponsor or person

Institute

Tohoku University

Institute

Department

Personal name

Funding Source

Organization

None

Organization

Division

Category of Funding Organization

Other

Nationality of Funding Organization

Other related organizations

Co-sponsor

Name of secondary funder(s)

IRB Contact (For public release)

Organization

N/A

Address

N/A

Tel

N/A

Email

N/A

Secondary IDs

Secondary IDs

NO

Study ID_1

Org. issuing International ID_1

Study ID_2

Org. issuing International ID_2

IND to MHLW

Institutions

Institutions

Other administrative information

Date of disclosure of the study information

2024 Year 06 Month 02 Day

Related information

URL releasing protocol

Publication of results

Unpublished

Result

URL related to results and publications

Number of participants that the trial has enrolled

Results

Results date posted

Results Delayed

Results Delay Reason

Date of the first journal publication of results

Baseline Characteristics

Participant flow

Adverse events

Outcome measures

Plan to share IPD

IPD sharing Plan description

Progress

Recruitment status

No longer recruiting

Date of protocol fixation

2024 Year 06 Month 01 Day

Date of IRB

2024 Year 06 Month 01 Day

Anticipated trial start date

2024 Year 06 Month 01 Day

Last follow-up date

2024 Year 12 Month 31 Day

Date of closure to data entry

Date trial data considered complete

Date analysis concluded

Other

Other related information

A search will be conducted using the following database and search terms, and two independent reviewers will be conducted a scre
will be conducted by a third reviewer as needed.
[Databases]
PubMed
[Search word]
'persistent critical illness', 'chronic critical illness', 'chronically critically ill', 'chronic critically ill', 'prolonged critical illness'
[Data Extraction]
Author(s), title, journal, year of publication, country, purpose, methods (measurement and analysis), participants (number and affiliati
question.

Management information

Registered date

2024 Year 06 Month 02 Day

Last modified on

2024 Year 11 Month 20 Day

Link to view the page

Value
https://center6.umin.ac.jp/cgi-open-bin/ctr_e/ctr_view.cgi?recptno=R000062304

Research Plan	
Registered date	File name

Research case data specifications	
Registered date	File name

Research case data	
Registered date	File name

Back

UMIN Infrastructure for Academic Activites
University hospital Medical Information Network
大学病院医療情報ネットワーク
